# Machine learning-enabled wastewater-based surveillance for emerging pathogen detection and monitoring: current applications, challenges, and future prospects

**DOI:** 10.1080/22221751.2026.2731499

**Published:** 2026-09-07

**Authors:** Anchao Song, Panpan Jia, Jing Luo, Xiaoli Pang, Yongqun He, Yi-Wei Tang

**Affiliations:** a College of Public Health, Chongqing Medical University, Chongqing, People’s Republic of China; b Department of Neurosurgery, University-Town Hospital, Chongqing Medical University, Chongqing, People's Republic of China; c Department of Laboratory Medicine and Pathology, University of Alberta, Edmonton, Canada; d Center for Computational Medicine and Bioinformatics, University of Michigan Medical School, Ann Arbor, MI, USA

**Keywords:** Wastewater-based surveillance, wastewater-based epidemiology, machine learning, early warning, predictive modelling, public-health decision support

## Abstract

Wastewater-based surveillance (WBS) has become an important public-health tool for tracking community-level circulation of emerging and re-emerging pathogens, but wastewater measurements are not directly interpretable public-health indicators. Signals recovered from sewer systems are shaped by sampling variability, environmental and inter- and intra-laboratory variations, population-dependent bias, and high-dimensional molecular complexity. Machine learning (ML) is increasingly used to extract predictive and decision-relevant information from these data, but the strength of the supporting evidence varies substantially. This narrative review examines how strongly current applications are supported by published evidence. Sources were identified through iterative searches of three literature-search sources updated to June 2026 and classified by role, analytical task, and strength of evidence. To keep the analytical scope consistent, we use a pragmatic four-layer classification: classical statistical models, conventional machine learning, deep learning, and hybrid or mechanistically informed approaches. Applications are reviewed across five analytical tasks: predictive modelling and forecasting, anomaly and fluctuation detection, data harmonization and normalization, high-dimensional interpretation, and integrated decision support. Among 35 sources reporting analytical or modelling work, 25 analyzed SARS-CoV-2 alone; demonstrated early-warning evidence came predominantly from L1 methods, and most target–task combinations remain prospective. Even within SARS-CoV-2 surveillance, reported performance was context-dependent. We examine the data-quality, methodological, and implementation barriers that limit broader application and validation. Future priorities include standardized and collaborative frameworks, multimodal data fusion, mechanistic and digital-twin modelling with explicit uncertainty assessment, and transparent and actionable analytics, with broader surveillance applications constrained by governance and public acceptability.

## Introduction

### Wastewater-based surveillance

Wastewater-based surveillance (WBS) has become an important public-health tool for monitoring infectious diseases and other population-level health indicators. Wastewater acts as a data-rich matrix that aggregates biological signals from entire communities, providing a non-invasive and cost-effective means of assessing pathogen circulation and community health status (Figure 1) [1]. By analyzing pathogens and biomarkers shed into sewer systems, WBS can provide near-real-time information on disease dynamics at the population scale [2–4].

**Figure 1.**
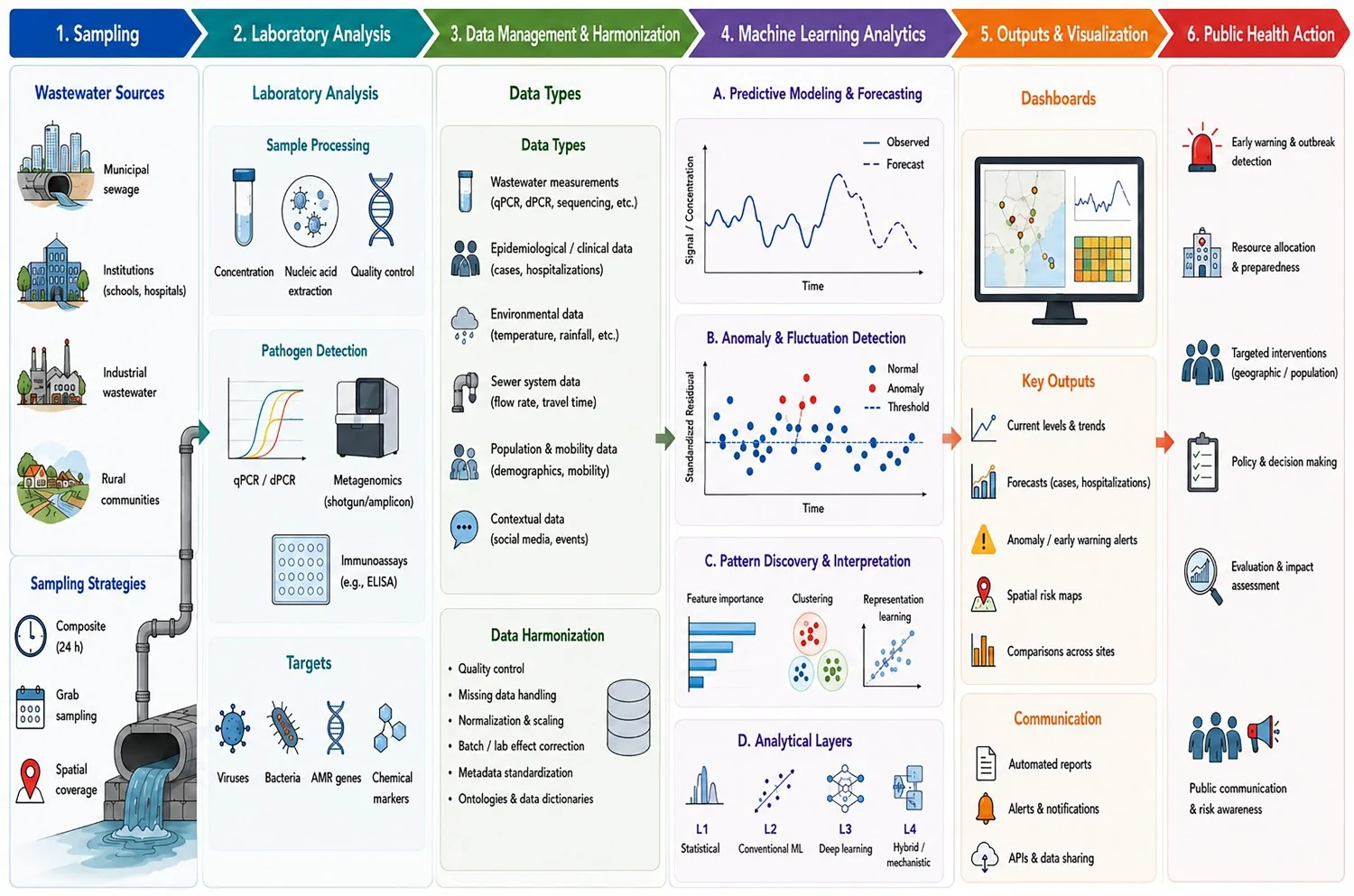
Workflow of machine learning-enabled wastewater surveillance. The framework comprises six interconnected stages (1) sampling of wastewater from diverse sources using appropriate strategies; (2) laboratory analysis including sample processing and pathogen detection; (3) data management and harmonization of multi-source heterogeneous data; (4) machine learning analytics for predictive modelling, anomaly detection, and pattern discovery; (5) generation of outputs, visualization, and communication; and (6) translation of insights into public health actions. Abbreviations: qPCR, quantitative polymerase chain reaction; dPCR, digital PCR; ELISA, enzyme-linked immunosorbent assay; AMR, antimicrobial resistance; ML, machine learning.

#### Historical development and surveillance evidence

Historically, WBS played an important role in poliovirus surveillance and global eradication efforts by enabling the detection of community transmission before clinical cases were recognized [5,6]. In London in 2022, 118 genetically linked type 2 polioviruses were recovered from 21 of 52 sequential sewage samples over five months, all of which had lost both principal attenuating mutations; no paralytic case was identified in the United Kingdom throughout this period [7]. More recently, widespread implementation during the COVID-19 pandemic demonstrated its value as a complementary surveillance tool capable of monitoring community-wide SARS-CoV-2 circulation, including infections that remained asymptomatic or unreported [8–10]. This under-ascertainment can be substantial: in Catalonia, the proportion of infections captured by clinical reporting was estimated to have fallen from about 40% during pre-Omicron waves to 19% after testing policy narrowed to severely symptomatic patients [11].

#### Institutionalization and data integration

Today, WBS has expanded beyond emergency pandemic response into long-term surveillance systems, supported by coordinated international investment in wastewater surveillance infrastructure [12]. Programmes such as the National Wastewater Surveillance System (NWSS) of the United States Centers for Disease Control and Prevention (CDC) [13,14] and the European Wastewater Surveillance Dashboard [15] provide continuous population-level monitoring and support public-health decision-making. Wastewater-derived data are increasingly integrated with epidemiological, environmental, and clinical information to support forecasting, outbreak investigation, and risk assessment (Figure 2; see the section on current applications).

**Figure 2.**
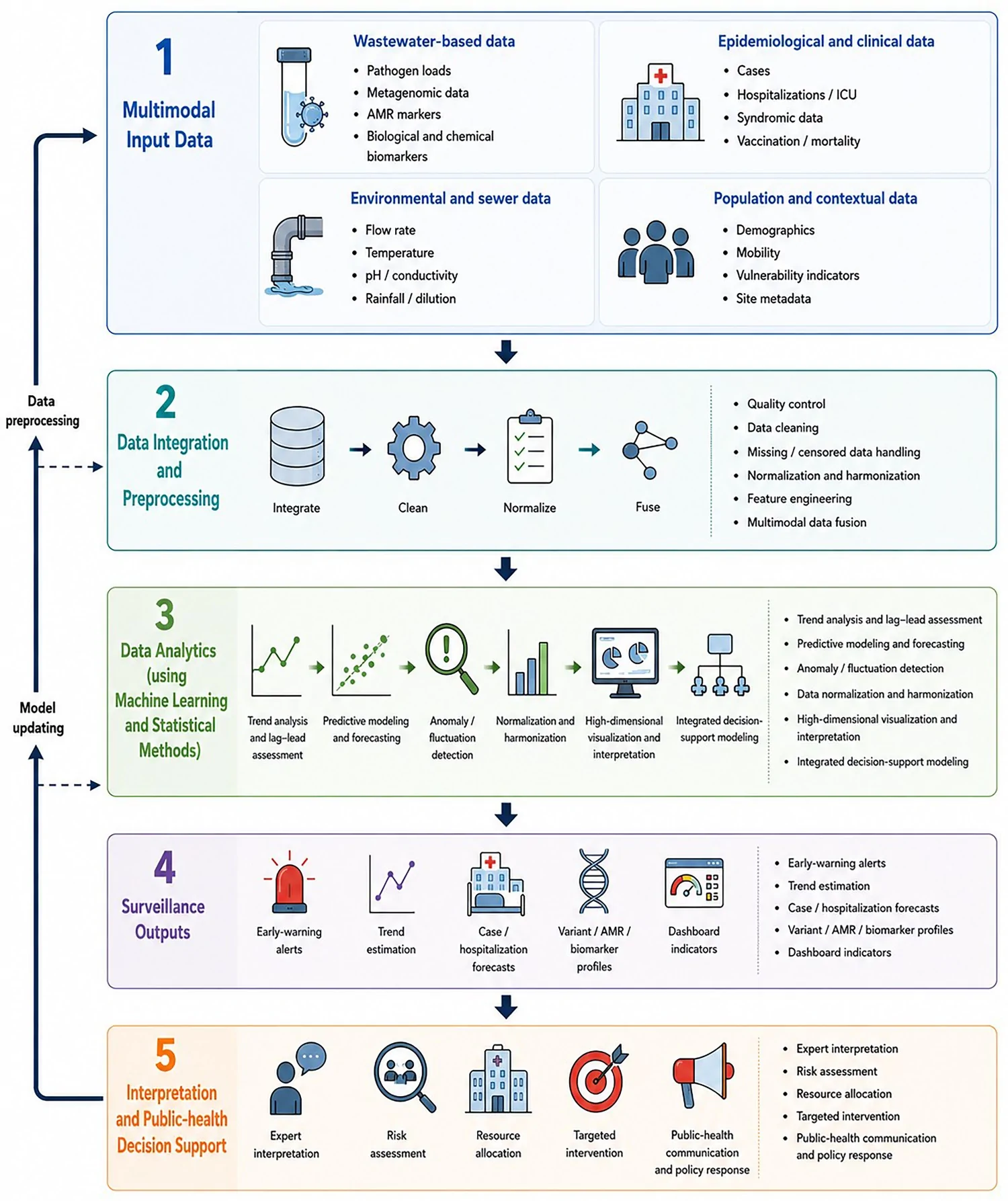
Multimodal data integration and translation to public-health decision support in machine learning-enabled wastewater-based surveillance (WBS). Multiple wastewater, epidemiological, clinical, environmental, sewer-system, population, and contextual data streams are integrated and harmonized before analysis using statistical and machine-learning approaches. Analytical outputs are transformed into surveillance indicators, including early-warning alerts, trend estimates, forecasting results, and variant/AMR profiles, which support public-health interpretation, risk assessment, resource allocation, targeted interventions, and policy decision-making. Continuous feedback enables data reprocessing and model updating.

#### Public-health value and practical limits

Several characteristics make WBS particularly valuable for public-health surveillance. First, wastewater signals can provide early-warning information before increases in reported cases or hospitalizations become evident, although reported lead times vary with pathogen, catchment, and clinical reporting practice, and part of the apparent lead reflects delay in clinical reporting rather than earlier biological detection [9,16–18]. Second, WBS captures community-level disease trends with less dependence on individual healthcare-seeking behaviour and testing capacity [19,20]. Third, advances in molecular technologies now allow simultaneous monitoring of multiple pathogens, antimicrobial resistance determinants, and other health-related biomarkers from a single sample [21–23]. These characteristics position WBS as an important component of integrated public-health surveillance systems for emerging and re-emerging infectious diseases, although its practical value depends on sewer coverage, catchment characteristics, and analytical capacity (see the sections on data quality and availability and on real-world implementation).

### Emerging pathogens and pandemic preparedness

Although WBS gained widespread visibility during the COVID-19 pandemic, its applications now extend well beyond SARS-CoV-2. Wastewater surveillance is being applied to a diverse range of viral, bacterial, parasitic, and antimicrobial resistance targets [21,22], supporting broader efforts in outbreak preparedness, One Health surveillance, and pandemic intelligence.

#### Respiratory, enteric, and One Health surveillance

Recent studies have demonstrated wastewater monitoring of respiratory viruses, including influenza A viruses, respiratory syncytial virus (RSV), enterovirus D68, and SARS-CoV-2 [24–27], as well as of enteric viruses, including noroviruses, rotavirus, and adenoviruses [27–29]. Wastewater surveillance has also been incorporated into monitoring of highly pathogenic avian influenza A(H5N1), providing complementary information to animal and clinical surveillance programmes during recent outbreaks [24]. Interpretation is constrained, however, wastewater testing does not by itself distinguish human from animal inputs. Among 24 sites with H5 detections, animal sources within or adjacent to the sewershed were identified at 15, including eight with milk-processing inputs [24]. RSV nucleic acids detected in wastewater have shown significant correlations with clinical positivity and hospitalization rates, supporting the feasibility of population-level RSV surveillance, although wastewater identified season onset earlier than clinical positivity in only 3 of 14 states and later in 7 [25]. Wastewater monitoring has also tracked an enterovirus D68 (EV-D68) outbreak, with wastewater viral loads and clinically diagnosed cases following a similar temporal course [26]. Because many emerging infectious threats originate at the human–animal–environment interface, wastewater surveillance may serve as an important One Health platform linking human, veterinary, and environmental surveillance systems [20,24].

#### Other emerging and re-emerging targets

Beyond respiratory viruses, WBS has also been applied to other emerging and re-emerging targets. Mpox virus DNA has been detected in wastewater in a pilot study spanning six US states, in which pre-amplification reduced false-negative detections by 87%. The authors described the approach as suitable for qualitative assessment of presence, but sensitivity for this low-titer target and standardization of detection methods remain unresolved [30]. WBS has also been applied to enteric and hepatitis viruses (Table 1) and to antimicrobial-resistant organisms [22], and has been proposed for novel influenza strains and other pathogens with epidemic or pandemic potential. Table 1 summarizes major emerging and re-emerging pathogens that have been detected through WBS; Table 2 then maps these targets onto the analytical tasks reviewed in the section on current applications and grades the evidence currently available for each combination.

**Table 1.** Major emerging and re-emerging pathogens detected in WBS.

Pathogen type	Pathogen	Major associated diseases	References
Viruses	SARS-CoV-2	Respiratory disease	[31]
	Influenza A virus (including H5 subtype)	Respiratory disease	[24]
	Respiratory syncytial virus (RSV)	Respiratory disease	[25]
	Enterovirus D68 (EV-D68)	Respiratory disease	[26]
	Norovirus	gastroenteritis	[27,28]
	Rotavirus	gastroenteritis	[28,32]
	Adenovirus	gastroenteritis, respiratory, ocular, and urological disease	[28,29]
	Hepatitis A virus (HAV)	Hepatic disease	[33]
	Hepatitis E virus (HEV)	Hepatic disease	[34]
	Mpox virus	Cutaneous disease; zoonotic disease	[30]
	Poliovirus	Neurological disease	[5–7]
Bacteria	*Campylobacter* spp.	Enteric disease	[35]
	*Salmonella* spp.	Enteric disease	[35]
	*Escherichia coli* (enteropathogenic)	Enteric disease	[36]
	*Shigella* spp.	Enteric disease	[35]
Parasites	*Cryptosporidium* spp.	Enteric disease	[37]
	*Giardia* spp.	Enteric disease	[37]
	*Entamoeba histolytica*	Enteric disease	[38]

Note: Species names are italicized. Table 2 maps these targets onto analytical tasks and grades the strength of the evidence available for each combination.

**Table 2.** Mapping of WBS targets to analytical tasks, with grading of the strength of currently available evidence.

Target	Detection and monitoring	Signal processing	Anomaly detection and early warning	Predictive modelling and forecasting	High-dimensional interpretation	Principal evidence gap
SARS-CoV-2	A [8,9,39,40]	A [27,41–45]	A [31,46–50]	A [11,17,18,51–57]; inconsistent or negative results reported [50,58,59]	A [55,60]	Reported performance has not been shown to transfer consistently across sewersheds, seasons, or laboratories [58,59]; most published models address temporal but not spatial structure [61].
Influenza A virus (including H5 subtype)	A [24]	C	B [62]	C	C	Wastewater influenza A signal may include non-human inputs [63]; early-warning performance is baseline-dependent, with wastewater preceding ILI-based onset only when the detection threshold rather than the annual geometric mean is used as the baseline [62]; no forecasting model has been validated against human incidence
Respiratory syncytial virus	A [25]	C	B [25]	C	C	Evidence is correlational; fecal shedding load and duration remain poorly characterized [64]; wastewater onset preceded clinical onset in only 3 of 14 states and lagged it in 7 [25]
Enterovirus D68	A [26]	C	C	C	C	Concordance with clinical surveillance shown qualitatively; no quantitative model
Norovirus, adenovirus, rotavirus	A [27–29,32]	C	C	C	C	Stable fecal shedding makes these favourable modelling targets, yet no prediction task has been attempted
Hepatitis A and E viruses	A [33,34]	C	B [33]	C	C	Low prevalence produces frequent non-detects; censored-data methods [44,45] untested for these targets
Mpox virus	A [30]	C	C	C	C	Short outbreak windows and low titres limit time-series modelling
Poliovirus	A [5–7]	C	A (programme level) [5–7]	C	C	Decades of programmatic early-warning use, but no published quantitative or machine-learning model
Enteric bacteria (*Campylobacter*, *Salmonella*, *Escherichia coli*, *Shigella*)	A [23,35,36]	C	C	C	C	Culture-independent quantification not standardized; no modelling study identified
Protozoan parasites (*Cryptosporidium*, *Giardia*, *Entamoeba histolytica*)	A [37,38]	C	C	C	C	Recovery efficiency highly variable; no modelling study identified
Antimicrobial resistance determinants and resistome	A [65–67]	C	C	C	B [65,66]	Deep-learning annotation operates on sequence data [68] rather than on wastewater time series; no forecasting of resistance burden reported
Community virome	A [60,69]	C	C	C	A [60,69]	Descriptive only; no established link between virome structure and clinical outcomes
Illicit drugs and other population-health biomarkers	A [70]	C	C	C	C	Standardization and generation of national estimates from multi-site data remain unresolved; no machine-learning application reported [71]

Note 1: **Evidence levels:** *A, empirically demonstrated* – at least one peer-reviewed study reports quantitative model output (predictions, classifications, or estimated indicators) for this target–task combination using wastewater data; *B, limited or single-setting evidence* – reported in a single programme, season, or region, or restricted to correlation and association without a fitted predictive model; *C, prospective only* – no published application to this target–task combination in wastewater, feasibility inferred from detection studies or from work in other domains. (Evidence levels were assigned from the quantitative results reported in the cited studies rather than from the authors’ self-described limitations.)

Note 2: **Analytical Tasks:** *Detection and monitoring* covers laboratory identification and quantification. *Signal processing* covers normalization, cross-laboratory harmonization, and handling of censored measurements. *Anomaly detection and early warning* covers trend-deviation detection, epidemic timing inference, and outbreak-risk classification. *Predictive modeling and forecasting* covers models fitted to estimate concurrent or future public-health indicators, such as case counts, hospital admissions, or infection prevalence, at a specified time horizon. *High-dimensional interpretation* covers dimensionality reduction, clustering, and explainable-model outputs.

#### Prospective detection of previously unrecognized pathogens

A further prospective application of WBS is the detection of previously unrecognized pathogens. For outbreak preparedness, the goal is to identify unusual biological signals before the underlying transmission is recognized as a disease cluster. Untargeted sequencing approaches that do not require prior knowledge of a specific target (see the section on laboratory methods), combined with advanced computational methods, could allow WBS to contribute to global pathogen intelligence systems for emerging biological threats and future pandemics (see the section on future prospects).

### Laboratory methods for pathogen detection and monitoring in WBS

Pathogen detection and quantification form the analytical foundation of WBS. Traditional surveillance approaches primarily rely on targeted molecular assays such as quantitative polymerase chain reaction (qPCR) and digital PCR (dPCR), which enable sensitive detection and quantification of known pathogens in wastewater samples [39,40,72]. These technologies underpin most of the detections summarized in Table 1 and remain the backbone of routine WBS programmes [3].

#### Sequencing strategies for broader pathogen detection

The scope of wastewater surveillance has expanded considerably with advances in next-generation sequencing technologies. Amplicon sequencing enables variant tracking and molecular epidemiology of specific pathogens. Shotgun metagenomic sequencing, by contrast, provides an untargeted approach capable of simultaneously characterizing viruses, bacteria, and antimicrobial resistance genes within a single sample [65,66,73]. Unlike targeted PCR-based methods, metagenomics does not require prior knowledge of specific pathogens and therefore offers opportunities for identifying unexpected or emerging infectious threats, although this breadth comes at the cost of sequencing depth on any individual target.

Between these two extremes lies probe-capture enrichment sequencing, also referred to as capture-based targeted next-generation sequencing (tNGS), in which probe panels are used to enrich sequences resembling known pathogens before sequencing. Because hybridization tolerates greater sequence divergence than primer binding, this approach retains sensitivity to variants that would escape amplicon assays, while increasing the relative abundance of its targets by orders of magnitude compared with untargeted shotgun sequencing of the same sample [74]. Comparative evaluation in wastewater has shown that deep shotgun metagenomic sequencing does not generate sufficient genome coverage of human pathogenic viruses for robust genomic epidemiology, whereas probe-capture enrichment substantially improves recovery of target genomes [73]. However, off-the-shelf panels have not yet recovered complete genomes for most non-enteric viruses [74]. In the same comparison, whole-genome tiled-PCR sequencing yielded higher coverage than hybrid capture for SARS-CoV-2, enterovirus D68, norovirus GII, and adenovirus F41 while requiring substantially less sequencing depth, and the authors identified it as the preferred method where the target is already known [73]. The advantage of capture therefore lies in the breadth of targets it can cover rather than in per-target performance. Panel composition is itself a constraint: in broad panels the reads are dominated by enteric viruses, and high-abundance targets consume sequencing capacity that would otherwise be available to low-abundance respiratory targets [74]. The approach is therefore best described as semi-targeted: it cannot detect pathogens absent from the probe panel, but it accommodates sequence variation within panel targets and can be extended as panels are redesigned. The wastewater virome study discussed below and in the section on visualization and interpretation of high-dimensional data was based on this approach [60]. Its outputs to date have supported pathogen detection, variant tracking, and virome characterization rather than predictive modelling, and no study has yet used capture-based sequencing data as an input to a predictive model in WBS (Table 2).

#### Viromic and resistome surveillance

Wastewater viromics applies sequencing-based surveillance at the viral-community level. By enriching and sequencing viral nucleic acids directly from wastewater samples, viromic approaches can characterize viral diversity, monitor viral evolution, identify emerging variants, and detect lineages of panel targets not previously reported in wastewater. In one longitudinal study of 363 samples, more than 450 pathogenic viruses from 28 viral families were identified, most of which had not previously been reported in wastewater [60]. Because enrichment is bounded by the probe set, however, such approaches broaden the range of viruses under routine observation rather than enabling untargeted pathogen discovery (see the section on emerging pathogens and pandemic preparedness).

Beyond viral surveillance, metagenomic sequencing is also used for antimicrobial resistance (AMR) surveillance. Community-level resistome profiling can characterize antimicrobial resistance genes, mobile genetic elements, and resistant bacterial populations circulating within a catchment area, thereby extending AMR surveillance beyond clinical settings and supporting One Health approaches to resistance monitoring [65,66].

#### Computational interpretation of sequence data

The increasing use of sequencing technologies generates large and complex datasets that require advanced computational approaches for interpretation. Machine-learning-assisted resistome analysis has been used to annotate antimicrobial resistance genes in metagenomic data with lower false-negative rates than alignment-based approaches [68]. These applications operate on sequence data rather than on the wastewater time series that are the principal subject of this review, and are considered separately in the section on visualization and interpretation of high-dimensional data.

### Data analysis in WBS: statistical and machine-learning approaches

Although pathogen detection is fundamental to WBS, wastewater measurements are not directly interpretable public-health indicators. Wastewater signals are influenced by numerous technical, environmental, and population-related factors, including sampling variability, rainfall-driven dilution, sewer-system characteristics, sample transport and storage conditions, PCR inhibition, and differences in population contribution to sewer networks [39,40,46,72]. Analytical methods are therefore needed to distinguish true epidemiological changes from background variability and measurement noise.

#### Statistical and machine-learning approaches as a continuum

Traditional statistical approaches remain central to trend analysis, correlation assessment, smoothing, uncertainty estimation, and early-warning interpretation. Modern WBS, however, increasingly generates heterogeneous and high-dimensional datasets that combine pathogen measurements with clinical, demographic, environmental, hydraulic, mobility, and genomic information, and such data have motivated the use of methods able to accommodate many correlated inputs at once [75].

Machine learning (ML) extends this analytical capacity by handling multimodal inputs, fitting nonlinear relationships, and building predictive models from large heterogeneous datasets [51,75]. In WBS, ML can integrate pathogen concentrations obtained through qPCR, dPCR, and sequencing-based methods with contextual information to support predictive modelling, anomaly and fluctuation detection, data harmonization and normalization, high-dimensional data interpretation, and integrated decision support (see the section on current applications). Where surveillance programmes incorporate metagenomic sequencing, wastewater viromics, or resistome profiling, the computational methods involved operate on sequence data rather than on wastewater time series, as noted in the section on laboratory methods, and are treated separately in the section on visualization and interpretation of high-dimensional data.

Statistical and machine-learning methods are best understood as points on a methodological continuum rather than as sharply separate categories; the distinction has long been described as one of modelling culture rather than of algorithmic substance [76]. Logistic regression, for example, is formulated as a generalized linear model but is also routinely used as a supervised classifier. Similarly, clustering-validation indices such as the silhouette coefficient originate in statistical pattern analysis yet are standard components of machine-learning workflows. To keep the analytical scope of this review consistent, we use a pragmatic four-layer classification that distinguishes classical statistical models, conventional machine learning, deep learning, and hybrid or mechanistically informed approaches.

#### Four-layer analytical framework

Layer 1 (L1), classical statistical models, impose a predefined statistical structure and are used mainly for description, inference, trend estimation, and signal detection; examples in WBS include correlation and lag–lead analysis, rolling averages, online trend estimation, Bayesian smoothing, and censored-data models. Layer 2 (L2), conventional machine learning, learns from data using shallow, structured learners such as regularized regression, tree ensembles (random forests, gradient boosting), clustering, and kernel methods. Layer 3 (L3), deep learning, learns hierarchical representations directly from sequences or high-dimensional inputs, using architectures such as deep neural networks, long short-term memory networks, and gated recurrent units. Layer 4 (L4), hybrid and mechanistically informed approaches, couple data-driven components with explicit epidemiological, hydraulic, or physical structure, and include semi-mechanistic Bayesian models, phenomenological neural networks, physics-informed learning, and digital twins [77].

#### Data requirements, combined workflows, and evidence scope

The four layers differ in data requirements, model flexibility, and computational demands. Many L1 methods can be fitted to the wastewater series itself, whereas supervised L2 and L3 models generally require paired outcome labels and larger training sets; L4 approaches additionally require mechanistic or system-level information. Computational and maintenance demands also tend to increase with model complexity; the corresponding trade-offs are summarized in Table 3. Throughout the sections on current applications and challenges, methods are therefore identified by analytical layer and these methods are treated as components of a single analytical workflow. A representative example is a hospitalization-forecasting study that first used correlation analysis to assess lagged associations (L1) and then applied random forest models for multivariable prediction (L2) [17].

**Table 3.** Analytical approaches used in WBS, organized by methodological layer, with data requirements, validation design, applicability boundaries, reported failure modes, and computational cost.

Layer	Method or model family	Typical WBS application and principal strength	Data requirements	Validation approach	Applicability boundary	Reported failure modes	Computational and deployment cost	Ref.
**Layer 1 – Classical statistical models (model structure specified a priori; inference and quality control)**								
**L1**	Correlation and lag–lead analysis	Characterizing temporal association between wastewater signals and clinical indicators; estimating lead time	Paired wastewater and clinical series; no training labels beyond the outcome series	Report correlation at multiple lags with confidence intervals; replicate across sites and seasons	Describes association only; cannot generate multivariable predictions or adjust for confounding	Lead time varies by outcome, site, and season, so a single pooled estimate can be misleading	Low	[17,31,78]
	Online trend estimation and deviation detection	Near-real-time identification of local departures from broader wastewater trends at sub-sewershed scale	Single wastewater series; no paired clinical labels required	Prospective evaluation on held-out periods; report false-alarm rate alongside detection timeliness	Detects change, not its cause; sensitivity depends on baseline window length	Sensitive to abrupt changes in sampling frequency or laboratory method, which mimic true deviations	Low	[47]
	Bayesian smoothing and outlier identification	Short-term projection, signal smoothing, and flagging of implausible measurements	Wastewater series with sampling dates; prior specification	Posterior predictive checks; assess interval coverage on held-out points	Short projection horizon; not designed for multivariable forecasting	Predictions deviated systematically at periods of rapid growth and decline and required recalibration	Low to moderate	[48]
	Left-censored and non-detect models	Unbiased trend estimation when measurements fall below limits of detection or quantification; site-specific model parameters can additionally flag sampling locations that contribute inconsistent or low-information data	Series with reported detection and quantification limits; censoring indicators	Compare with substitution approaches; sensitivity analysis over the non-detect fraction	Requires that censoring limits be reported; performance degrades as the non-detect fraction rises	Ignoring censoring distorts trend estimates during low-prevalence and non-endemic periods; different non-detect handling methods yield materially different wastewater–clinical agreement on the same dataset	Low to moderate	[44,45,79]
	Normalization and cross-laboratory harmonization	Adjustment for dilution, population size, and fecal load; alignment of units, metadata, and laboratory methods	Flow, population estimates, or endogenous indicators (PMMoV, crAssphage, RNase *P*); split-sample data for inter-laboratory comparison	Compare normalization schemes against clinical reference within the same dataset; split-sample inter-laboratory testing	Optimal scheme is site-specific; no single normalizer performs best across all settings	Normalization does not uniformly improve correlation or lead time; indicator choice materially changes results, and RNase *P* performed less well than PMMoV or CrAssphage across catchment sizes.	Low	[27,41,42,49,80,81]
**Layer 2 – Conventional machine learning (structure learned from data using shallow learners)**								
L2	Regularized and (generalized) linear regression baselines	Benchmark predictor against which more complex models are judged	Paired wastewater and outcome series; modest sample size	Rolling-origin or blocked cross-validation; compare against naive persistence	Limited capacity for non-linear or interaction effects	Performance depends strongly on whether variant-related features are included	Low	[54]
	Random forest	Forecasting weekly hospital admissions and case counts from wastewater combined with clinical, weather, vaccination, and vulnerability covariates	Paired outcome labels; multiple covariates; at least one full epidemic season of regular (typically weekly) observations	Leave-one-site-out and rolling-origin validation; external validation across catchments and years	Requires paired clinical labels; extrapolates poorly beyond the covariate range seen in training	Performance highly sensitive to regional data structure, site heterogeneity, and forecasting context across 108 sites in five regions	Moderate	[17,53,54,58]
	Gradient boosting with time-aware feature engineering	Nowcasting and forecasting case dynamics using site-specific lagged wastewater and environmental features	Paired outcome labels; dense series permitting lag construction; feature-selection step	Strict temporal splits with a buffer between training and test periods to prevent leakage	Lag structure is site-specific and may not transfer; tuning burden is substantial	Gains over a naive baseline were site-, horizon-, and metric-dependent: across two sites the short-term improvement in RMSE ranged from 21% to 0.02%, and some feature configurations failed to beat the naive baseline for forecasts. Boosted trees fitted without temporal structure were outperformed by time-aware models on the same dataset.	Moderate	[51,52]
	Clustering and classification	Outbreak-risk classification and surge-week detection translated directly into alert categories	Wastewater loads plus contextual covariates; labelled surge and inter-surge periods	Held-out period classification accuracy at one- and two-week horizons	Categorical output only; cluster labels may lack stable epidemiological meaning across sites or periods	Classification performed well (87.9% within-period; 80.4% and 81.8% at one and two weeks ahead) while regression on weekly case counts in the same dataset remained limited	Low to moderate	[50]
	Quantile regression for anomaly flagging	Post-laboratory screening of routinely reported measurements to flag values inconsistent with comparable sites	Multi-site concentration series with site metadata (geography, collection method); no outcome labels required	Compare flagged values against known operational events and against measurements from comparable sites	Flags inconsistency relative to peer sites, not epidemiological change; requires a sufficient number of comparable sites	Screening identified 762 anomalous results among 25,957 samples reporting no quantified value; anomalies clustered in periods of high national incidence, indicating that low values can arise from sampling or laboratory factors rather than low prevalence	Moderate	[46]
	Dimensionality reduction and visualization (clustering and t-SNE, L2; PCA and ordination, L1)	Exploration of metagenomic, virome, and resistome data; revealing separation by site and sampling date	High-dimensional molecular data; no outcome labels required	Assess stability across parameter settings and random seeds	Exploratory; embeddings do not support quantitative prediction	Visual summaries can oversimplify complex community interactions and lead to misinterpretation of the underlying structure; some tools also impose computational demands that limit access where high-performance computing is unavailable	Moderate	[50,60,65,66,82]
**Layer 3 – Deep learning (hierarchical representations learned from sequences or high-dimensional inputs)**								
L3	Deep sequence and feedforward models (LSTM, DNN)	Multi-community outbreak prediction and temporal forecasting across multiple sites	Large labelled multi-site datasets; long and regular time series	Temporal cross-validation with held-out sites and periods; compare against L1 and L2 baselines	WBS series are typically too short for the data requirements of these architectures; GRU-based designs remain prospective in WBS; the cited evidence comes from a multi-model comparison that also included statistical (Prophet) and tree-based learners on the same dataset, rather than from standalone deployment	Evidence not quantified in the cited study for this failure mode	High	[52]
	Deep learning for sequence annotation	Prediction of antibiotic resistance genes from short reads and full-length sequences in metagenomic data	Reference databases and metagenomic sequence data; no wastewater time series required	Benchmark against alignment-based annotation on held-out reference sets	Operates on sequence data, not on wastewater time series; does not forecast resistance burden	Depends on the coverage and quality of the underlying ARG databases; several resistance categories are absent from the source databases, and multidrug-category assignments required manual inspection.	High	[68]
**Layer 4 – Hybrid and mechanistically informed approaches (data-driven components coupled with explicit structure)**								
L4	Semi-mechanistic Bayesian generative models	Joint modelling of heterogeneous multi-site wastewater measurements with hospital-admission data; probabilistic forecasting with explicit uncertainty	Multi-site wastewater and outcome data; laboratory and site metadata; prior specification	Assess calibration and interval coverage, not point accuracy alone; evaluate by site and by period	Computationally intensive; conclusions depend on structural and prior assumptions	Wastewater inputs did not improve forecasts uniformly across locations and time periods	High	[59]
	Mechanism-informed neural networks	Estimation of unobserved (silent) infections from national wastewater surveillance data with improved interpretability relative to generic architectures	Large-scale surveillance data; an explicit phenomenological or epidemiological structure	External validation against independent epidemiological indicators	Inference of unobserved quantities cannot be verified directly from wastewater alone	Estimates inherit the bias of the assumed shedding and transmission structure	High	[56]
	Digital twins and simulation-based frameworks	Coupling wastewater measurements with epidemiological, hydraulic, and operational models for scenario testing and model recalibration	Sewer-network and hydraulic data; real-time sensing; calibrated mechanistic models	Validate against observed wastewater and hydraulic behaviour, including known failure scenarios such as storm dilution	Currently demonstrated for validation and operational control rather than for causal inference	Long-horizon performance depends on the quality of online catchment data and rainfall forecasts, with uncertainty becoming particularly important during high-flow conditions [83].	High	[11,83,84]^†^
**Cross-cutting – applied to models at Layers 2 to 4**								
–	Explainable AI (SHAP and related attribution methods)	Identification of influential predictors; communication of model behaviour to public-health users	A fitted model plus its training data; no additional data collection	Compare attributions across methods and across resampled datasets for stability	Explains model behaviour, not causal effect; attributions can be unstable under correlated predictors	Does not improve predictive performance; explanations may vary among methods and can oversimplify interactions	Low to moderate	[55,85]^†^

Note: Layers are defined in the section on data analysis in WBS as a pragmatic classification of classical statistical models, conventional machine learning, deep learning, and hybrid or mechanistically informed approaches; they are complementary components of a single analytical workflow. This table characterizes methods; Table 2 grades the strength of evidence available for each pathogen–task combination. Data requirements state the minimum conditions under which the approach has been applied in the cited studies. Validation approach follows the time- and space-aware designs discussed in the section on methodological complexities; random k-fold cross-validation is not appropriate for autocorrelated wastewater series. Reported failure modes are drawn from quantitative results reported in the cited studies rather than from the authors’ self-described limitations. Computational and deployment cost is graded qualitatively as low (executable on standard hardware within routine reporting pipelines), moderate (requires dedicated computation and periodic retraining), or high (requires substantial computing resources, specialist staff, and continuous maintenance). ^†^Supported in part by evidence from outside wastewater-based surveillance. Digital twins have so far been demonstrated in wastewater treatment and sewer-network operation [83,84]; the only surveillance application identified is [11]. Attribution methods are reviewed here from digital health [85]; the only wastewater application identified is [55].

Beyond forecasting established indicators such as case counts and hospital admissions, ML-enabled WBS has been proposed as a means of supporting earlier recognition of emerging pathogens, identifying unusual transmission patterns, and recognizing novel variants or resistance mechanisms. How far each of these possibilities is supported by published evidence varies markedly by pathogen and by analytical task, and is assessed in the section on current applications (Table 2). Figure 3 summarizes the major challenges that constrain ML-enabled WBS and the corresponding future directions.

**Figure 3.**
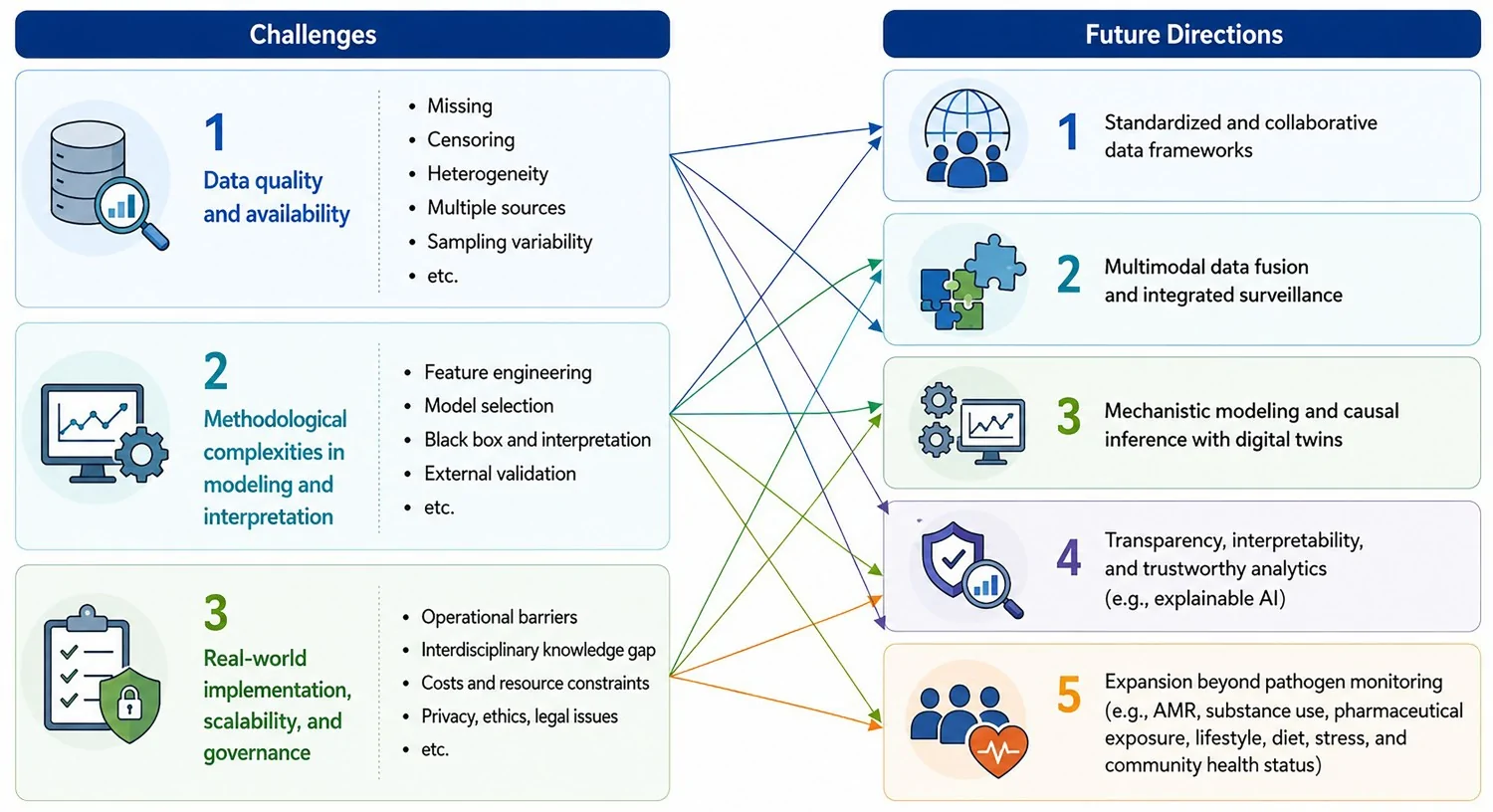
Challenges and future directions of machine learning in wastewater-based surveillance (WBS). Major challenges for machine learning-enabled WBS include limitations in data quality and availability, methodological complexities in model development and interpretation, and barriers to real-world implementation, scalability, and governance. Future directions focus on standardized data frameworks, multimodal data integration, digital-twin-based mechanistic modelling, explainable and trustworthy analytics, and expansion of WBS applications beyond pathogen surveillance to broader population-health monitoring. Arrows indicate the interrelationships between current challenges and potential future solutions.

### Literature search and selection strategy

This article is a narrative review rather than a systematic review, and no formal quality scoring or quantitative synthesis was undertaken. For transparency, we summarize how the cited sources were identified, selected, and categorized, in line with item 3 of the SANRA scale, which requires narrative reviews to report their literature search [86]. The full procedure is given in the Supplementary Methods.

#### Search strategy and source classification

Sources were identified through iterative searches of PubMed, Web of Science Core Collection, and Google Scholar, with the final update on 30 June 2026. The searches combined terms for the surveillance setting, the analytical approach, the surveillance target, and implementation and governance, and were supplemented by citation tracking and by targeted consultation of national surveillance programmes and standards bodies (Supplementary Table S1). Each cited source was assigned to one of six roles: background and scope; pathogen detection and monitoring; analytical evidence; methodological reference; implementation and governance; and programme infrastructure and standards. Sources providing analytical evidence were further classified by analytical task and by methodological layer, with the layer assigned from the methods actually reported rather than from the authors’ own description of their approach (see the section on data analysis in WBS). The composition of the cited literature is summarized in Supplementary Table S2, and the role assignment and citing sections for every individual source are listed in Supplementary Table S3.

#### Composition and imbalance of the evidence base

The resulting evidence base spans 2001–2026, with 104 of 125 sources (83%) published in 2020 or later. The evidence base is broad in pathogen detection but narrow in analytical modelling: 33 sources provide evidence on pathogen detection and monitoring across a broad range of viral, bacterial, parasitic, and antimicrobial-resistance targets. By contrast, among the 35 sources reporting analytical or modelling work, 25 analyze SARS-CoV-2 alone, four include it among several targets, and only six address targets other than SARS-CoV-2. This asymmetry reflects the available published evidence and motivates our grading of the strength of evidence for each target–task combination in Table 2 and our calibration of the conclusions in the Summary to the breadth and strength of that evidence.

#### Search limitations and retention of negative evidence

This strategy has three limitations. Screening and categorization were performed by the authors without dual independent review; screening was restricted to English-language sources, which may under-represent work reported in other languages and compound the geographic concentration described in the section on real-world implementation; and studies reporting unsuccessful or inconsistent model performance are likely to be under-represented in the published record. We have therefore deliberately retained and explicitly discussed reported negative, null, and inconsistent findings [27,50,58,59] rather than presenting only successful applications.

The sections that follow use the four-layer pragmatic classification defined above and calibrate conclusions to the strength of evidence available for each target–task combination. The section on current applications reviews five analytical tasks – predictive modelling and forecasting, anomaly and fluctuation detection, data harmonization and normalization, high-dimensional interpretation, and integrated decision support – and distinguishes demonstrated from prospective evidence across surveillance targets. The section on challenges examines the principal constraints on these applications, including data quality and availability, methodological complexity, and real-world implementation, scalability, and governance. The section on future prospects considers future development through standardized and collaborative frameworks, multimodal data fusion, mechanistic and digital-twin modelling with explicit uncertainty assessment, transparent and actionable analytics, and extension beyond pathogen monitoring. The Summary sets out the evidence, its limitations, and the implications for future development. Three tables organize the synthesis from complementary perspectives: Table 1 lists surveillance targets detected through WBS; Table 2 maps these targets to analytical tasks and grades the evidence for each target–task combination; and Table 3 compares analytical approaches by methodological layer, data and validation requirements, applicability, reported failure modes, and computational demands.

## Current applications of machine learning in WBS

Machine learning (ML) is increasingly used in WBS to analyze complex wastewater measurements alongside other data sources. ML can extend conventional statistical analysis by integrating multiple data streams, modelling nonlinear and lagged relationships, and detecting abnormal signal changes [46,51,75]. This section summarizes current ML-related applications in WBS across predictive modelling, anomaly and fluctuation detection, data harmonization and normalization, high-dimensional data interpretation, and integrated decision support.

The evidence underlying these applications is unevenly distributed across pathogens. Of the 35 sources reporting analytical or modelling work, 25 analyze SARS-CoV-2 alone and four more include it among several targets (Supplementary Table S2). Several features of the COVID-19 data environment help explain this concentration. During the COVID-19 pandemic, wastewater programmes operated under a combination of conditions that rarely coincide: high community prevalence, dense and sustained sampling, increasingly standardized assays, and paired clinical case and hospitalization data. Together, these conditions are unusually favourable for supervised modelling; for most other targets, one or more are weaker or absent. For many other pathogens, prevalence is low enough that a substantial proportion of measurements fall below limits of detection or quantification. Shedding magnitude, duration, and route differ considerably among pathogens and remain poorly characterized for influenza viruses, respiratory syncytial virus, and human metapneumovirus [64]. Partition coefficients between the liquid and solid phases of wastewater vary by orders of magnitude across viruses, so an identical sample-processing protocol yields very different recovery depending on the target [87]. For influenza A, the wastewater signal may additionally include non-human inputs, so measured concentrations cannot be interpreted as a proxy for human prevalence without source attribution [63]. Model performance reported for SARS-CoV-2 should therefore not be assumed to transfer to other pathogens. Table 2 maps the pathogens introduced in Table 1 onto the analytical tasks reviewed in this section and grades the strength of the evidence available for each combination; most combinations remain prospective. The published record may also over-represent successful applications. We have therefore retained studies reporting limited, inconsistent, or negative results [27,50,58,59] and discuss them alongside positive findings throughout this section.

### Predictive modelling and forecasting

Predictive modelling in WBS spans statistical, data-driven, and mechanistically informed approaches, with performance depending strongly on the available data and forecasting context.

#### Statistical baselines and early-warning relationships

Predictive modelling and forecasting are among the most developed analytical tasks in WBS. Models in this category are fitted to forecast public-health indicators such as hospitalization rates and case counts. In Austria, cross-correlation analysis (L1) was used to quantify the time lead of wastewater signals over hospital bed occupancy across 123 treatment plants, and multivariate regression models – ordinary least squares and support vector regression, each with and without ridge or lasso regularization (L1–L2) – were then compared for inferring occupancy [18]. The authors report that forecast models were sensitive to the circulating variant and required recalibration [18]. As set out in the section on data analysis in WBS, traditional lag–lead and correlation-based analyses (L1) can already characterize early-warning relationships in some WBS settings [17,78], and remain the only analytical approach reported for several non-SARS-CoV-2 targets (Table 2).

#### Data-driven and hybrid forecasting

Where the available inputs include multiple heterogeneous covariates, however, learned models can capture nonlinear and lagged structure without strict parametric assumptions. Li et al. employed random forest models (L2) trained on county-level wastewater data, hospitalization records, vaccination coverage, weather variables, and community vulnerability indices from 159 U.S. counties. The resulting models forecasted weekly new COVID-19 hospital admissions one to four weeks in advance [17]. Lai et al. developed an interpretable time-series machine-learning (TSML) framework to nowcast and forecast COVID-19 case dynamics using wastewater and environmental variables [51]. The framework combined gradient boosting machines (L2) with feature engineering and feature selection to incorporate site-specific temporal relationships between wastewater signals and reported cases, supporting nowcasting and forecasting across several horizons. Against a naive baseline, the improvement ranged from 21% at one site to negligible at the other [51]. Ai et al. compared time-series and non-time-series learners across nine sewersheds and found that the time-series-aware models, Prophet (L1) and long short-term memory (LSTM) networks (L3), outperformed gradient-boosting decision trees and deep neural networks fitted without temporal structure; the authors describe the work as a proof of concept requiring further real-world deployment [52]. Ribalta et al. fitted extreme-gradient-boosting models (L2) to weekly wastewater and case data from 56 treatment plants in Catalonia and used SHapley Additive exPlanations to identify the variables driving the predictions; catchment population and the N gene indicator were consistently the most influential. The models tracked the direction of change in case counts but underestimated peak magnitude, and the authors attribute part of the residual error to weekly rather than near-daily sampling [55].

Hybrid and mechanistically informed approaches (L4) have also been applied to SARS-CoV-2: a phenomenological neural network trained on National Wastewater Surveillance System data was used to estimate unreported infections [56], and a digital twin of transmission in Catalonia used sewage data to validate model predictions and to support long-term forecasts [11]. Other studies have reported forecasting with random forests and SARIMAX models in Latvia, the United States, Liechtenstein, and Austria [53,54,57]. Across these studies, the forecasting evidence remains overwhelmingly SARS-CoV-2-specific. Whether comparable performance can be achieved for pathogens with sparser signals and fewer paired clinical labels has not been tested.

#### Performance limits and context dependence

Reported performance has also been inconsistent. Vaughan et al. evaluated random-forest time-series forecasting (L2) across 108 sites in five regions and found accuracy to depend chiefly on sampling frequency and training-set size, with the contribution of catchment population less clear [58]. In a semi-mechanistic Bayesian forecasting model (L4) for state-level hospital admissions, adding wastewater data left overall performance essentially unchanged, although it improved or degraded forecasts substantially at particular locations and dates [59]. Model complexity is therefore not a benefit in itself: predictive performance depends on sampling frequency, training-set size, location, and forecasting context, and adding wastewater data does not improve forecasts uniformly.

### Anomaly and fluctuation detection for early warning

The task of anomaly and fluctuation detection differs from forecasting. Rather than estimating the future value of a public-health indicator, it identifies unexpected spikes, shifts, or departures from baseline trends that may signal an emerging outbreak [47–50]. The methods used for this purpose span the first two layers defined in the section on data analysis in WBS and can be combined.

#### Statistical detection and signal quality

At the statistical layer (L1), Ensor et al. applied online trend estimation and deviation detection to sub-sewershed SARS-CoV-2 RNA time series, providing a near-real-time framework for identifying local departures from broader wastewater trends [47]. Because the model is fitted to the series itself, it requires no paired clinical labels and can be updated as each new measurement arrives, allowing it to be incorporated into routine reporting pipelines. Related L1 procedures and quality-control measures address the same problem from the data-quality side: Bayesian smoothing supports short-term projection and outlier identification [48], and routine quality-control indicators, in particular flow rate and gene-segment ratios, help separate measurement outliers from genuine concentration changes across more than 150 treatment plants [49]. Measurements below the limit of quantification require separate treatment, because substituting a fixed value biases both the estimated concentration and its trend. Two censored-data smoothers developed for this purpose – an autoregressive smoother robust to outliers, in operational use by the French national network, and a left-censored dynamic linear model – were compared under cross-validation with each other and with a simple moving average using data from 286 sewage treatment works in England. Both tracked regional case-rate positivity more closely than untransformed data or the moving average; at comparable predictive accuracy, the dynamic linear model was more parsimonious and faster to compute [45,79]. In an early-warning system, distinguishing a measurement artifact from a genuine epidemiological change is part of the detection task.

#### Machine-learning classification and anomaly detection

At the conventional machine-learning layer (L2), Armenta-Castro et al. developed clustering- and classification-based risk-assessment models using wastewater viral loads from treatment plants and university campuses, mobility data, and clinically reported COVID-19 cases [50]. Their clustering model distinguished surge weeks from inter-surge periods with 87.9% accuracy and maintained 80.4% and 81.8% accuracy for one- and two-week-ahead classification, respectively. Multivariable linear regression (L1) fitted to the same 48 weekly observations predicted weekly case numbers poorly, indicating that, in this dataset, low-dimensional WBS data may support outbreak-risk classification more reliably than precise case-count forecasting. At the same layer, the English national programme trained a gradient-boosting regression model with a quantile loss function to predict 90% concentration intervals at each sampling site. The lower bound of the interval was then used to flag measurements that were anomalously low relative to comparable sites; 762 anomalous results were identified among 25,957 samples that reported no quantified value [46].

#### Operational evaluation and method choice

For early warning, performance should be judged by timeliness of detection and false-alarm rate rather than by point-prediction accuracy alone; a model that identifies surge onset correctly may be operationally more useful than one with a lower forecasting error. The choice of analytical method should also be justified by demonstrated performance rather than assumed from its complexity. A general time-series benchmark of 1,045 monthly series found that widely used machine-learning methods were outperformed by established statistical methods across all horizons examined while requiring considerably greater computation [88]. This provides a useful caution for WBS, where demonstrated early-warning results have come predominantly from L1 methods: across 12 long-term care facilities monitored for 28 months, wastewater signals led 32 of 53 confirmed outbreaks and lagged only 12, with non-detection acting as a negative predictor of outbreak [78]; in a national analysis of 189 treatment plants across 40 states, cross-correlation indicated that wastewater concentrations preceded hospital admissions by 2–12 days [31]; and online trend estimation and Bayesian smoothing have supported deviation detection at sub-sewershed and municipal scale [47,48]. Evidence for L2 and L3 methods, by contrast, has been concentrated mainly in multivariable forecasting in which clinical and contextual covariates are available [17,51,52]. An L2–L4 approach should therefore be adopted only when it improves a pre-specified operational metric over both a naive persistence baseline and the best available simpler alternative, evaluated under the validation designs discussed in the section on methodological complexities.

### Data harmonization and normalization

Data harmonization and normalization support the analytical applications discussed in this section because reliable modelling, forecasting, and anomaly detection depend on measurements that are consistent and comparable across sites and over time. These operations are mainly upstream of model fitting and include unit conversion, metadata alignment, adjustment for dilution and population size, and correction of laboratory-specific bias; where statistical adjustment is involved, it falls mainly within L1. Wastewater data are nonetheless particularly difficult to harmonize, because they are multi-source and heterogeneous, combining pathogen concentrations with hydraulic, environmental, laboratory, and population-level information.

#### Data standards and harmonization

At a broader data-structure level, comparability across programmes depends not only on harmonizing individual variables but also on organizing data according to shared principles and controlled vocabularies. The FAIR principles define four properties of reusable data: Findable, Accessible, Interoperable, and Reusable, with explicit emphasis on machine-actionability as well as on reuse by individual researchers [89]. Ontologies, which standardize and logically represent entities and relations within a domain, have become central to biomedical data integration, sharing, and validation [90], and interoperable ontologies support data FAIRness by providing a structured semantic framework [91]. In the wastewater field, an ontology-based environmental decision-support system (OntoWEDSS) has been reported, although it was developed for treatment-plant operation rather than for epidemiological surveillance [92].

Harmonization focuses on aligning multi-source WBS datasets across sites, laboratories, sampling frequencies, measurement units, metadata structures, and reporting systems. Without such alignment, jointly analyzing wastewater, clinical, environmental, and demographic variables can introduce systematic bias, weaken comparability, and reduce model transferability. Therrien et al. introduced the Public Health Environmental Surveillance Open Data Model (PHES-ODM), an open-source data model and metadata framework for wastewater-based epidemiology that supports interoperable data collection and cross-programme harmonization of environmental surveillance datasets [80]. Chik et al. analyzed split-sample results from 15 laboratory methods across 15 rounds in Canada and showed that method-specific biases can be quantified from inter-laboratory comparisons. SARS-CoV-2 measurements showed good concordance across laboratories, whereas PMMoV measurements did not, and PMMoV normalization did not improve method comparability [41]. The authors caution that the method-specific coefficients derived in this way can correct relative bias only where laboratory procedures are otherwise comparable [41]. Reporting conventions also affect comparability: different methods for handling qPCR non-detects yield materially different agreement between wastewater and clinical data, so the method used should be specified in reporting [44].

#### Normalization and context dependence

Normalization approaches typically involve adjusting pathogen concentrations using flow rates, population estimates, or endogenous human fecal indicators, such as PMMoV, crAssphage, and RNase P, to compensate for rainfall-driven dilution, population-size variation, and fecal-load variability [27,42,43]. These indicators are not interchangeable. Across 650 samples from four Kentucky counties, RNase P was less useful than PMMoV or crAssphage when catchment size varied, and the daily sewer flow at the sampling point mattered more than rainfall for interpreting indicator concentrations [43]. The evidence on whether normalization improves epidemiological correspondence is, however, mixed. Maal-Bared et al. compared normalization by flow, population size, and biomarkers across ten communities in twelve Alberta sewersheds (n = 1024) and found that PMMoV normalization significantly improved correlations in only two systems, neither large nor served by combined sewers; in the pooled dataset no normalized concentration was significantly more strongly associated with case numbers than the unnormalized concentration, and the authors recommend applying at least two normalization approaches in parallel [42]. Darling et al. reported that in a small rural sewershed with substantial inflow and infiltration, physicochemical normalizers introduced additional variability and yielded signals more closely associated with precipitation than with pathogen concentrations, whereas human fecal markers remained comparatively robust [27]. Together, these findings suggest that measuring flow at the point of sampling may contribute more to interpretable normalization than adding a molecular indicator, and that in smaller treatment plants investment in flow metering may be of greater value than routine PMMoV testing [42,43].

A national implementation shows what combining these corrections can achieve. The French wastewater indicator, which applies the autoregressive censored smoother described in the section on anomaly and fluctuation detection to each series, additionally accounts for the different limits of quantification used by individual laboratories, adjusts for inflow variation, and provides inter-laboratory normalization where inter-laboratory assays are unavailable; measurement noise fell by roughly one-fifth across 168 plants, and in the seven plants with matched incidence data the correlation with reported incidence improved by 18% relative to raw PCR results [81]. The effect of normalization is therefore context-dependent rather than uniformly beneficial. Its value depends on sewer characteristics, on the choice of indicator, and on whether laboratory-method differences and censored measurements are addressed at the same time.

#### Implications for downstream modelling and transferability

After harmonization and normalization, comparable wastewater inputs can be integrated with clinical, environmental, and demographic variables for downstream modelling. Model transferability depends on consistent measurement units, metadata structures, and preprocessing pipelines [17]. Wastewater-informed forecasting frameworks show that heterogeneous measurements from multiple sites and laboratories can be jointly modelled with hospital-admission data, although the same work shows that wastewater inputs do not always improve forecasts uniformly across locations and time periods [59]. Harmonization and normalization therefore affect downstream model performance. Model flexibility cannot compensate for inputs that are not comparable.

### Visualization and interpretation of high-dimensional WBS data

Dimensionality-reduction and clustering methods support the visualization and interpretation of high-dimensional WBS data. The increasing use of metagenomics, viromics, and resistome profiling generates datasets whose volume and complexity make direct interpretation difficult, and a range of visualization approaches has been developed to make such data explorable by non-specialists [82]. Principal component analysis and related ordination methods (L1), together with clustering and non-linear embeddings such as t-distributed stochastic neighbour embedding (t-SNE) (L2), reduce dimensionality, reveal sample groupings, and expose spatial or temporal structure in wastewater data. Their outputs are descriptive rather than predictive: they organize data for inspection but do not by themselves yield a public-health indicator.

#### Viromic pattern visualization

Tisza et al. combined probe-based viral capture with deep wastewater virome sequencing and applied t-SNE to viral abundance data; samples separated clearly by city of collection and by sampling date [60]. The embedding revealed spatial and temporal structure in viral-community composition that is not available from single-target assays. At a much smaller scale, the risk-assessment study discussed in the section on anomaly and fluctuation detection used principal component analysis and clustergram visualization to summarize patterns among weekly WBS observations [50].

#### Metagenomic and resistome interpretation

Similar strategies apply to sewage metagenomics and AMR monitoring, where high-dimensional resistome profiles can be converted into interpretable patterns for selecting surveillance targets and prioritizing follow-up analyses. Global sewage surveys have used principal component analysis and clustered heatmaps to visualize regional structure in resistance genes and bacterial genera [65], and non-metric multidimensional scaling with similarity testing has been used to compare resistome profiles across treatment plants and to identify the genes that best distinguish sample groups [66]. Across these applications, low-dimensional representations have been used to compare samples and communities, describe spatial and temporal structure, and guide the selection of surveillance targets, rather than to generate public-health forecasts (Table 2).

### Integrating WBS and epidemiological data: applications and design requirements

ML-enabled WBS should be embedded in an integrated surveillance system rather than treated as a standalone wastewater signal. Clinical case counts, syndromic reports, hospital and ICU admissions, vaccination coverage, serological status, pathogen genomic data, and demographic or contextual variables provide complementary information about disease burden, severity, immunity, and population vulnerability. WBS contributes population-level measurements that can capture asymptomatic and untested infections, but it cannot by itself distinguish shedding sources, individual risk, clinical severity, or intervention needs. Combining these streams can help public-health agencies interpret wastewater trends in relation to outbreak activity and population vulnerability while distinguishing epidemiological change from environmental noise [17,50].

#### Applications and decision-support roles

Examples discussed above illustrate two distinct uses of integration: forecasting and outbreak-risk classification. Li et al. combined county-level WBS data with hospitalization records, vaccination coverage, weather variables, and community vulnerability indices to forecast COVID-19 hospital admissions one to four weeks ahead (see the section on predictive modelling and forecasting) [17], whereas Armenta-Castro et al. integrated WBS, mobility, and clinical reports to translate weekly wastewater observations into outbreak-risk classifications for rapid local decision-making, while also showing that low-dimensional datasets limit precise regression forecasts (see the section on anomaly and fluctuation detection) [50].

In an integrated pipeline, wastewater signals can serve as predictors; clinical diagnoses and hospitalization data can provide outcome labels; environmental and operational variables can be used to account for dilution and sampling variability; and serology or vaccination data can contextualize the relationship between viral circulation and severe disease. Each of these roles carries an implicit assumption about how the variables are causally related, and those assumptions determine whether adjustment reduces or introduces bias. The aim is decision support rather than automated prescriptive guidance: models can provide risk scores, short-term forecasts, uncertainty intervals, explanatory features, and alert levels for interpretation by epidemiologists and public-health officials.

#### Causal structure and shortcut learning

Integrating additional data streams, however, does not by itself remove bias; it can introduce it. Meteorological, hydraulic, mobility, and clinical variables are not independent covariates but sit within a causal structure that determines whether adjusting for them reduces or introduces bias. Three configurations recur in WBS. First, rainfall can affect the measured wastewater signal through dilution and changes in flow, while also influencing human movement and transmission, thereby creating a backdoor path between the measured wastewater signal and reported cases. Second, clinical testing intensity influences reported case counts without influencing wastewater shedding, so models trained against reported cases may learn features of surveillance behaviour rather than of transmission. Third, season affects rainfall, in-sewer decay, and pathogen circulation simultaneously, so associations between environmental covariates and wastewater signals may reflect shared seasonality rather than direct causal effects.

Configurations of this kind create the conditions for shortcut learning, in which a model achieves high accuracy on data drawn from the training distribution by exploiting a proxy that has no stable relationship to the outcome outside it [93]. Such shortcut learning is one mechanism of the poor cross-region transferability discussed in the section on methodological complexities. A model that has learned to track testing intensity or a site-specific rainfall pattern will fail precisely when it is transferred to a catchment where those patterns differ.

#### Prediction, causal estimation, and explicit assumptions

The analytical objective should therefore be stated explicitly, because prediction and causal estimation impose different requirements on variable selection [94]. For prediction, variables can be selected on the basis of out-of-sample performance, provided that they are available at the time the prediction is made. For questions framed causally, such as whether an intervention changed transmission or whether a wastewater rise reflects genuine spread rather than a reporting artifact, the adjustment set must follow the assumed causal structure: confounding paths should be blocked, whereas conditioning on mediators or colliders can change the estimand or introduce bias [94].

The assumed causal structure should also be made explicit. Representing the assumed structure as a directed acyclic graph (DAG) before modelling makes the adjustment set explicit and auditable, and established tools can enumerate minimal sufficient adjustment sets [95]. Figure 4 illustrates such a structure for a typical multimodal WBS pipeline. In our search, we identified no published WBS modelling study that reported an explicit causal structure of this kind. Making the assumed structure explicit would improve the transparency of model documentation.

**Figure 4.**
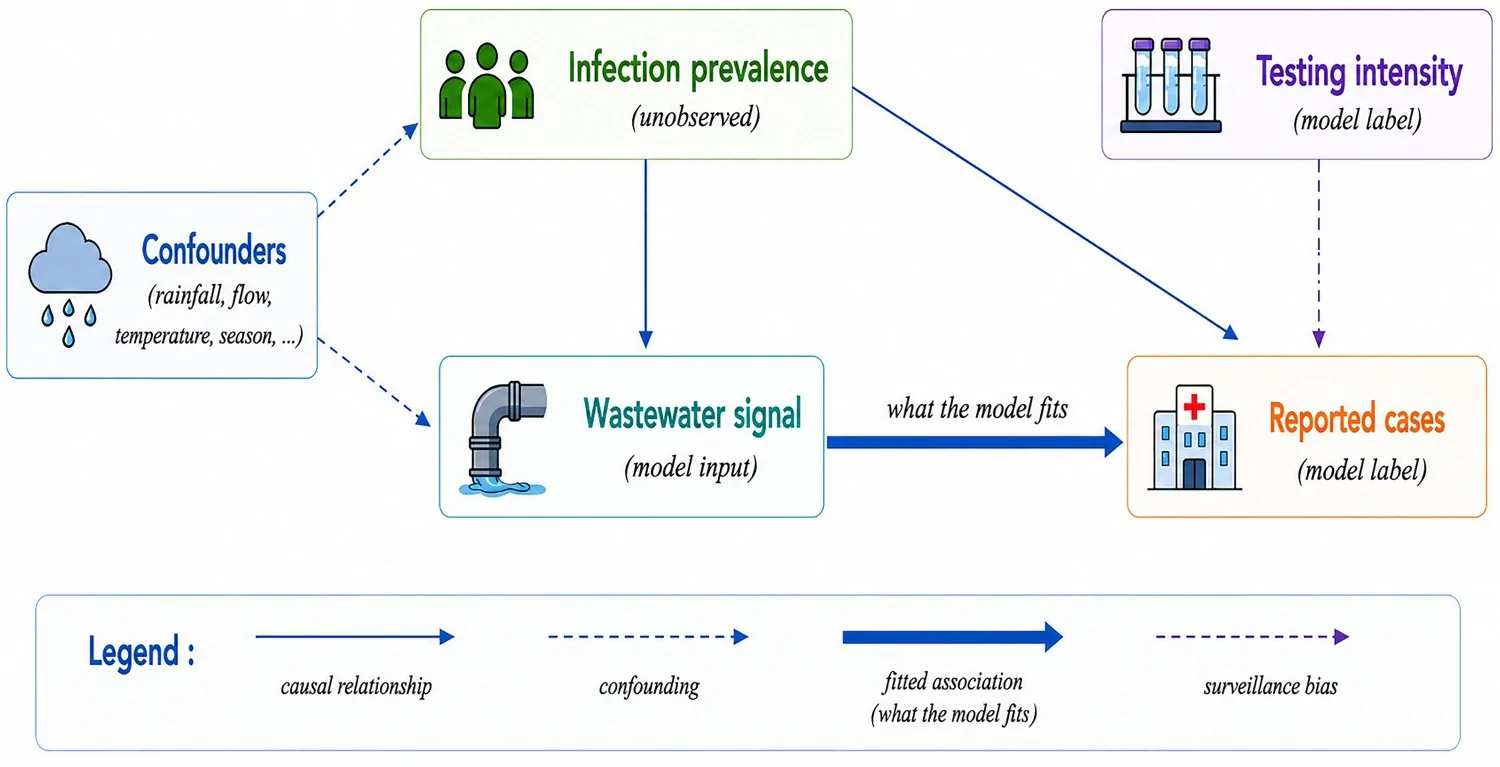
Assumed causal structure underlying multimodal wastewater-based surveillance modelling. Community infection prevalence is not directly observed but causes both the measured wastewater signal and reported cases; the association that a predictive model actually fits (thick arrow) is therefore not itself causal. Environmental and seasonal variables, represented here as potential confounders, may act as common causes of both endpoints of that association: rainfall can affect the wastewater signal through dilution and changes in flow while also influencing human movement and transmission, while season and temperature can additionally affect in-sewer decay and pathogen circulation. Such variables may therefore open backdoor paths that should be addressed when the analytical objective is causal, but they may legitimately remain in a predictive model when the objective is prediction alone. The dash-dotted arrow denotes surveillance bias: testing intensity affects reported cases without affecting shedding, so a model trained against reported cases may partly learn features of surveillance behaviour rather than of transmission. This graph is illustrative; the appropriate structure is pathogen- and setting-specific and should be stated explicitly for each analysis.

To consolidate the method-specific evidence discussed above, Table 3 summarizes representative analytical approaches used in WBS by the four methodological layers defined in the section on data analysis in WBS, together with their typical applications, data requirements, validation designs, applicability boundaries, reported failure modes, and computational costs. Methods from different layers can be combined within a single workflow: classical statistical analyses, tree-based learners, Bayesian models, and explainable-AI tools may be applied sequentially or in combination to the same dataset, as illustrated by the studies reviewed above.

## Challenges in applying machine learning to WBS

Practical use of ML in WBS is constrained by challenges across the full workflow, from sampling and preprocessing to model training, validation, interpretation, and deployment. These challenges generally fall into three categories: data quality and availability, methodological complexities in modelling and interpretation, and implementation and scalability in real-world public-health systems. These constraints affect not only technical performance but also interpretability, transferability, operational feasibility, and responsible public-health use.

### Data quality and availability

High-quality input data are essential for building robust ML models, yet WBS data present several upstream data-quality challenges. These uncertainties propagate into ML models, reducing prediction accuracy, model transferability, and the reliability of downstream public-health interpretation. They do not propagate uniformly: the same data limitation can be minor for one model family and severely distorting for another, so the consequences of data-quality problems should be considered in relation to the model family being used.

#### Censoring and low-signal measurements

Data missingness and censoring are common in WBS. During early outbreaks or low-prevalence periods, pathogen concentrations may fall below limits of detection or quantification, and the analytical platform itself shapes how often this occurs: in low-prevalence catchments in Queensland, RT-dPCR achieved limits of detection two to five times lower than RT-qPCR and significantly higher detection rates in both the liquid and solid fractions of the same samples [39]. Methods for handling these low-end observations were introduced in the sections on anomaly and fluctuation detection and on data harmonization and normalization [44,45]. What follows from them for machine learning depends on how censored values are handled and on which model family is applied.

Two distinct problems are often conflated under the heading of censoring. A non-detect is treated as indicating that no target was observed and the true concentration lies somewhere between zero and the limit of detection, whereas a measurement below the limit of quantification indicates that the target was detected but cannot be assigned a reliable value. The former is an interval-censored observation, the latter a measurement with inflated error, and treating both as identical discards information. How they are handled matters more as their frequency rises: during early outbreaks, in low-prevalence periods, and for pathogens with low shedding loads, censored measurements can constitute a substantial fraction of a series rather than an occasional inconvenience.

The consequences of this choice are not uniform across model families. The differences follow from how each family uses its inputs rather than from evidence specific to wastewater, and they have received little attention in this setting. Tree-based learners (L2) split on rank rather than magnitude, so the numerical value assigned to a non-detect matters less than the fact that a single substituted constant collapses all censored observations onto one point; the learner may then treat that constant as an informative category rather than as absent information. Linear and neural-network models (L2 and L3) are sensitive to the substituted magnitude itself, so the choice between zero, half the detection limit, and the detection limit divided by the square root of two can systematically alter the fitted relationship. Sequence models (L3) are affected in another way: a run of consecutive non-detects is presented as a genuine low plateau, which can inflate apparent stability during exactly the low-prevalence periods when early warning matters most. Substitution has long been shown to distort summary statistics and to obscure trends in environmental data, and to be capable of reversing conclusions about whether an association exists [96]; these effects propagate into any downstream model fitted to the substituted series.

On methodological grounds, these considerations suggest a practical hierarchy of approaches, although direct WBS-specific comparisons across model families remain unavailable. Explicit censored-data models, such as the left-censored dynamic model applied to SARS-CoV-2 wastewater series [45], retain the information that a measurement fell below a threshold without assigning an arbitrary value and should be considered when censoring limits are available. Multiple imputation provides an alternative when censored-data likelihoods are impractical, provided that the resulting uncertainty is propagated into the downstream model rather than discarded after a single completed dataset is produced. Single substitution has been argued to be generally unacceptable in scientific reporting [96]; where it is nonetheless used, it should be confined to series in which censored observations are infrequent and accompanied by a sensitivity analysis across plausible substitution values, since a conclusion that changes across that range is sensitive to the substitution choice and should not be treated as robust. Reporting requirements follow directly: the detection and quantification limits, the proportion of censored observations, and the handling strategy should be stated for every wastewater series used as a model input, since none of these can be inferred from a concentration time series alone. In our search, we identified no study that systematically compared censoring-handling strategies across model families using wastewater data, and the resulting uncertainty about which combinations amplify rather than reduce bias remains an unresolved methodological gap. The expected model-family-specific consequences of censoring and substitution, set out as analytical expectations rather than empirical findings, are summarized in Supplementary Table S4.

#### Measurement and catchment heterogeneity

Data heterogeneity also hinders model development and generalization. Differences in sampling strategies, analytical platforms, and normalization approaches can introduce systematic biases that limit data comparability [27,39,97,98]. Not all such differences are equally consequential, however. Länsivaara et al. found that the RNA extraction kit, the RT-PCR kit, and the target gene assay each influenced measured concentrations and had to be optimized jointly, while also identifying isothermal amplification as a qualitative detection option for low-resource settings [97]. Augusto et al., by contrast, compared grab and 24-hour composite sampling at two treatment plants and a sewer manhole over 17 weeks and found no significant difference in mean SARS-CoV-2 concentrations between the two designs (Pearson r = 0.83), with within-day coefficients of variation between 3.0% and 11.5% [98]. The effect of sampling design on comparability is therefore context-dependent and requires evaluation.

Catchment-specific differences in geography, sewer infrastructure, flow rate, pH, inhibitors, industrial discharges, and in-sewer processes can likewise alter wastewater properties and pathogen signal persistence, making it difficult to develop models that generalize across diverse regions [27,99]. Li et al. showed in a sewer reactor that biofilms play two roles in the fate of SARS-CoV-2 RNA: they mediate in-sewer decay, and they act as a reservoir that accumulates RNA and releases it back into the wastewater under changing hydraulic conditions [99]. The estimated RNA loss was small in systems with high prevalence but became consequential in low-case settings and over long hydraulic residence times, which are the same conditions under which censored measurements are most frequent. WBS data quality is therefore affected not only by laboratory procedures but also by upstream sewer processes whose effects become most consequential when the signal is weakest.

#### Structural limitations in data availability

Limited data availability is a further constraint, and it arises in two distinct ways: the population of interest may not contribute to the sewer, or the target may not be circulating at detectable concentrations. Van den Berg et al. assessed ten child daycare centres in the Netherlands for wastewater surveillance and found none suitable, because the centres were embedded in mixed-use or multi-tenant buildings and installing dedicated sampling points was judged disproportionately demanding in resources and permissions [100]. The same study notes a second and separate limitation, that diaper-wearing children do not use sanitary facilities and therefore do not contribute directly to the wastewater stream, so that even a feasible sampling point would under-represent the population of interest. The second constraint appears when the target itself is scarce: Casado-Martín et al. monitored SARS-CoV-2, influenza A and B, and RSV-A and RSV-B in a mid-sized Spanish city over twelve months and found that SARS-CoV-2 concentrations correlated with reported cases (r > 0.5), whereas influenza A and B were not detected, RSV-B was detected in only two samples, and RSV-A circulation declined under non-pharmaceutical interventions [101]. Data availability in WBS is therefore both pathogen- and population-dependent, and a run of non-detects may reflect under-representation of the population of interest, an unsuitable sampling point, or genuinely absent circulation – distinctions that a concentration time series alone cannot resolve.

### Methodological complexities in modelling and interpretation

Translating noisy and heterogeneous WBS inputs into accurate, interpretable, and transferable ML models remains a major methodological challenge. Model development may involve feature engineering across multiple data streams, such as pathogen concentrations, hydraulic and meteorological variables, clinical reports, vaccination records, and demographic or contextual indicators, while accounting for non-stationarity, lagged relationships, and distributional shifts. These tasks require interdisciplinary expertise to select relevant variables, construct time-aware and biologically plausible features, and validate models under designs that respect the dependence structure of the data.

#### Model selection and feature design

Model selection further complicates ML-enabled WBS, because no single model family is consistently optimal across surveillance objectives and data settings. Depending on the task, candidate models span all four layers defined in the section on data analysis in WBS, from correlation and regression models to tree ensembles, recurrent architectures, and semi-mechanistic frameworks. The studies reviewed in the section on predictive modelling and forecasting show that performance depends on both the data available and the model design. Vaughan et al. found that forecast accuracy depended chiefly on sampling frequency and training-set size, with the contribution of catchment population less clear [58]; Dejus et al. found that performance depended on both algorithm choice and the inclusion of variant-related features [53]; and Ai et al. found that models incorporating temporal structure outperformed those fitted without it on the same dataset [52]. Model selection in ML-enabled WBS is therefore not merely a choice among algorithms; it also depends on feature availability, prediction horizon, geographic transferability, interpretability, and the public-health objective. Geographic transferability remains particularly underexamined.

#### Cross-catchment transferability

Several recurring sources of cross-catchment variation can limit transferability. Sewer configuration and hydraulic residence time differ between combined and separate systems, and in-sewer biofilms alter biomarker persistence before a sample is collected, most strongly in low-prevalence settings and over long residence times (see the section on data quality and availability) [99]. Climate and rainfall regimes differ, so the relationship between measured concentration and community prevalence is modulated differently at each site. Laboratory methods differ in concentration, extraction, and quantification, and inter-laboratory split-sample comparison has shown that these differences produce measurable method-specific bias [41]. Population structure and sewer connectivity differ, so the fraction of a catchment represented in the sewer signal is itself site-dependent [100,101]. Together, these factors can alter both the distribution of model inputs and the relationship between wastewater measurements and epidemiological outcomes. Cross-catchment transfer should therefore be treated as a distribution-shift problem whose mechanism may differ among sites. A model that learns a site-specific proxy rather than a stable transmission signal may fail when transferred, as discussed in the section on integrating WBS and epidemiological data.

Different approaches address different sources of transfer failure. Site-specific intercepts or hierarchical structures allow each catchment to have its own baseline while sharing information across sites; semi-mechanistic Bayesian frameworks applied to multi-site wastewater and hospital-admission data implement this partial pooling explicitly [59]. At the feature level, normalization by flow or by endogenous fecal indicators can reduce site- and time-specific variation before modelling [27,42], while harmonized data models and metadata standards address variation at the level of data structure [80]. Transfer learning and domain adaptation provide methods for adapting information learned under one data distribution to another [102]. In our search, we identified no study evaluating these methods for cross-catchment transfer in WBS, so their value in this setting remains untested. Finally, where data-sharing agreements rather than statistical structure are the binding constraint, federated learning allows multiple laboratories or agencies to train a shared model without exchanging raw data; the approach has been developed for clinical and imaging data rather than for wastewater [103], and it addresses governance barriers rather than distributional ones.

#### Validation under temporal and spatial dependence

Whether such transfer failures are detected depends on how models are validated, and WBS data pose a structural difficulty. Wastewater concentration series and the epidemic curves they track are both strongly autocorrelated: consecutive observations are statistically dependent and often highly similar rather than independent draws, so a randomly partitioned series can place temporally adjacent observations in both the training and test sets, and the resulting error estimate may partly reflect interpolation between nearby observations and can overstate genuine forecasting performance. A related problem arises across space, since neighbouring catchments share climate, mobility patterns, and often laboratory methods. Empirical evidence on how far this inflates reported accuracy is mixed: in a comparison of six model-selection procedures on synthetic and real-world series, no systematic under- or overestimation of error was attributable to the dependencies themselves, although blocked cross-validation yielded more robust error estimates than reserving the final segment of each series [104]. In ecological modelling, where spatial and phylogenetic dependence is pervasive, blocked designs have been shown to be preferable to random partitioning wherever the goal is prediction to new data or new predictor space, with the caveat that blocking can itself induce extrapolation by restricting the predictor combinations available for training [105]. Neither literature source is specific to WBS, but both indicate that the partitioning scheme should follow the dependence structure of the data rather than convenience.

Several validation and reporting practices follow. Temporal evaluation should use rolling-origin or blocked designs in which every test period falls strictly after its training period, with a gap where needed to reduce dependence across the training–test boundary; where the model is intended for transfer, spatial evaluation should be blocked by site, with performance reported for held-out catchments rather than pooled. Any preprocessing step whose parameters are estimated from the data should be fitted within the training data only; applying data-dependent smoothing, normalization, or scaling to the full series can leak information from the test period. Reported performance should always be accompanied by the forecast horizon and by comparison against a naive baseline set out in the section on anomaly and fluctuation detection. Because censored observations are unevenly distributed over an epidemic cycle (see the section on data quality and availability), the proportion of non-detects in the training and test periods should also be reported, so that a decline in apparent performance is not misattributed to poor generalization when it reflects a change in the censored fraction. Few of the WBS modelling studies reviewed here report enough detail on validation design to establish whether temporal and spatial dependence were respected, which limits interpretation of their reported performance. More complete validation reporting is therefore needed. Recommended validation and reporting practices are summarized in Supplementary Table S5.

#### Interpretability and transparency

A further barrier to practical adoption is the opacity of some complex models, particularly those in L3, where predictive accuracy may not be accompanied by an interpretable account of how inputs relate to outputs. This matters in public health, where decision-makers require defensible evidence to justify interventions. A recent review of AI applications in wastewater epidemiology notes that some machine-learning and deep-learning approaches in this field are described as black-box systems, and that several studies have deliberately adopted regression- or tree-based models to make the basis of a prediction visible [106]. The phenomenological neural network discussed in the section on predictive modelling and forecasting illustrates a related strategy: it embeds explicit epidemiological structure, making its estimates of unreported infection more interpretable [56]. Model selection should therefore consider interpretability and transparency alongside predictive accuracy, while external validation is needed to establish whether performance transfers beyond the development data.

### Real-world implementation, scalability, and governance

The practical barriers to ML-enabled WBS can be grouped into operational integration, resource and infrastructure constraints, and governance and public acceptability.

#### Operational integration and interdisciplinary coordination

Operational barriers also limit the effective implementation and scalability of ML-enabled WBS. Model outputs must be translated into practical decision-support tools, such as risk thresholds, alerts, uncertainty summaries, and visual dashboards, that fit existing public-health workflows. Naughton et al. reviewed 127 SARS-CoV-2 wastewater dashboards from 27 countries and found high variability in presentation, including graph type, linear versus logarithmic scaling, and 96 separate ways of labelling wastewater concentrations; only 30% provided downloadable source data and 25% presented variant monitoring [107]. Inconsistent dashboard visualization and reporting can therefore limit cross-site comparability, weaken risk communication, and create scope for public-health misinterpretation. The risk-classification approach discussed in the section on anomaly and fluctuation detection provides one example of making analytical outputs operational, by converting weekly observations into local outbreak-risk categories rather than reporting regression accuracy alone [50]. Operational use therefore requires standard operating procedures, shared metadata, reporting pipelines, and regular data-quality checks [108,109].

Interdisciplinary coordination is another implementation challenge. Effective implementation of ML-enabled WBS requires cooperation across data science, microbiology, environmental engineering, wastewater utilities, epidemiology, and public health. Gitter et al., reporting on a statewide network in Texas, observed that practitioners in these sectors had few natural venues for working together. Two barriers emerged early: recruiting utilities required navigating legal agreements, sampling and shipping costs, and local political considerations that varied between municipalities; and a reporting process had to be developed before wastewater findings could support timely public-health action [110]. Bridging these gaps requires routine communication, shared metadata and data dictionaries, clear reporting expectations, and boundary-spanning individuals or teams that can translate between disciplines [108,110]. Without such capacity, ML outputs may be technically valid yet misinterpreted, underused, or disconnected from public-health practice.

#### Resource, infrastructure, and setting constraints

Costs and resource constraints can also hinder long-term implementation, particularly in low- and middle-income countries. Although WBS can be cost-effective relative to population-wide clinical testing, sustained high-coverage surveillance still requires stable investment in sampling infrastructure, sample transport, laboratory testing, data management, and trained personnel [111,112]. A survey of 12 wastewater monitoring programmes in low- and middle-income countries found per-sample costs ranging from USD 34 in India to USD 517 in Costa Rica, with a median all-inclusive cost of USD 185; recurring costs accounted for a greater share of annual expenditure than fixed costs, and the sampling approach alone explained 46% of the variation in per-sample cost [113]. Programmes that used composite sampling faced two- to three-fold higher per-sample costs than those using grab sampling from open trenches, pit latrines, and other non-centralized locations (median USD 465 versus USD 185), showing that sampling designs compatible with regular time-series monitoring may be substantially more expensive to sustain. Programmes monitoring larger populations, and those including rural or peri-urban communities rather than urban populations only, also reported lower per-sample costs. These findings show that financing affects which sampling designs a programme can sustain, and those choices in turn constrain the analytical methods that can be supported.

Infrastructure coverage creates a separate scalability constraint. All empirical ML applications identified in this review were developed using data from centralized municipal sewer networks in high-income settings, yet onsite sanitation systems such as pit latrines and septic tanks serve at least 1.8 billion people in low- and middle-income countries, and in most cities of the Global South sewers reach only a small proportion of the population and often do not extend to unplanned settlements where disease burden may be highest [114]. Surveillance in these settings relies on a wider set of matrices, including fecal sludge collected at disposal points, open drains, and impacted surface water, an approach often distinguished from sewer-based WBS as environmental surveillance [115]. These alternative matrices also change the structure of the data available for analysis. Catchment boundaries are often poorly defined, so the contributing population may be difficult to delineate; the denominator required for population normalization may therefore be uncertain, and flow-based normalization may be unavailable where flow is not measured. Pathogen concentrations differ systematically between matrices, by roughly two orders of magnitude in open drains and three in impacted river water relative to treatment-plant influent, and are sensitive to antecedent rainfall [115], so recovery efficiency and decay behaviour cannot be assumed to match those established for sewer influent. Sampling is often event-driven, tied to de-sludging schedules or rainfall, rather than producing the regular series that time-series models require. Some operational constraints may be less severe: sampling depth within pit latrines did not measurably affect the odds of detecting enteric pathogens, suggesting that a surface sample may be adequate for routine monitoring [114].

For machine learning, these differences challenge assumptions built into the dominant WBS modelling template. The dominant modelling template in WBS assumes a regularly sampled concentration series, a defined and normalizable population denominator, and paired clinical labels from the same catchment. In non-sewered settings none of these three assumptions holds reliably, so models developed under that template cannot be assumed to transfer directly without redefining both the input representation and the target variable. Plausible adaptations include treating detection as a binary or ordinal outcome rather than estimating concentration, modelling at the level of the sampling site rather than an inferred population, and borrowing strength across sites through hierarchical structures rather than fitting each site independently. In our search, we identified no published WBS study evaluating these adaptations, and the absence of such work means that the settings with the least clinical surveillance capacity, and therefore the greatest potential benefit from wastewater-derived intelligence, are also those for which the analytical toolkit is least developed.

#### Governance and public acceptability

Public trust remains foundational for sustainable WBS programmes, and broad adoption of ML-enabled WBS raises ethical, legal, and social questions that require proactive governance. Wastewater data are aggregated, but linking them with high-resolution demographic, mobility, or setting-specific data can raise group-privacy concerns. Ethics guidance developed for this field concluded that neither classical clinical bioethics nor general public-health ethics adequately addresses the issues raised by wastewater surveillance, and adopted the WHO guidelines on ethical public-health surveillance as the applicable framework [116]. A 2023 United States survey illustrates how acceptability varies by surveillance target and spatial scale: support for wastewater surveillance of disease and of toxins was high, at 91% for each, but fell to 49% for alcohol consumption, 46% for mental illness, and 35% for lifestyle behaviours; support was 85% for citywide surveillance and markedly lower for smaller, less anonymous geographic scales [117]. Acceptability is therefore conditional on both the target and the spatial resolution, which bears directly on proposals to extend WBS to non-infectious biomarkers (see the section on applications beyond pathogen monitoring) and to building- or sub-sewershed-level monitoring. A structured ethical review framework for wastewater-based testing has been proposed to make these considerations explicit, and its retrospective application to 53 SARS-CoV-2 monitoring programmes found that 43% of its questions could not be answered from the information those programmes reported [118]. A legal analysis similarly argues that policy action taken on the basis of a positive sewage signal requires careful attention to the scientific limitations of the measurement alongside its public-health benefits [119]. Transparent governance is therefore a prerequisite for responsible WBS, particularly where data support community-level behavioural inference.

Overall, the practical value of ML-enabled WBS depends on the surveillance system in which a model operates, not on algorithmic performance alone. Data quality, representativeness, interpretability, operational usability, and the fit between model assumptions and deployment conditions all affect whether outputs can support public-health decisions. Reliable implementation requires robust data pipelines, careful model validation, interdisciplinary collaboration, sustainable financing, and transparent governance. Meeting these requirements is necessary for sustained use of ML-based wastewater analytics in public-health decision support.

## Future prospects for applying machine learning to WBS

WBS has moved from emergency pandemic monitoring toward routine public-health surveillance, supported by molecular quantification, genomic and metagenomic sequencing, contextual metadata, and large-scale reporting infrastructures. Future development of ML-enabled WBS will depend on surveillance systems that are standardized, interoperable, multimodal, mechanistically informed, interpretable, ethically governed, and able to extend beyond pathogen-specific monitoring. The directions below are evidence-informed but not uniformly validated in WBS; they build on the findings reviewed in the sections on current applications and challenges, while Table 2 shows where the empirical evidence remains incomplete.

### Building an open, standardized, and collaborative framework

A mature WBS framework requires alignment across operational standards, data and analytical reporting, and cross-disciplinary coordination.

#### Operational and institutional standardization

The limitations identified in the preceding sections point to the need for open, standardized, and collaborative frameworks spanning the full operational chain of WBS, from sampling and laboratory processing to metadata harmonization, data sharing, reporting, and governance. Formal guidance and standards are already available or under development for sampling and laboratory procedures: WHO guidance on environmental surveillance for SARS-CoV-2 [3] and ISO standards under development for wastewater detection and quantification [109] support comparability and reproducibility across programmes. By contrast, reporting remains heterogeneous, as illustrated by the variability among national dashboards documented in the section on real-world implementation.

Two assessments by the National Academies of Sciences, Engineering, and Medicine have outlined the characteristics required for a mature wastewater surveillance system. The first envisages a national wastewater surveillance system that is flexible, equitable, sustainable, integrated, and actionable. Flexibility refers to the capacity to monitor multiple pathogens simultaneously and adapt rapidly to emerging threats; equity concerns representation and coverage across population groups, including efforts to extend surveillance capacity to communities without sewer connections; and integration refers to triangulation with other disease surveillance systems [120]. The second focuses on translating such a system into public-health action and gives particular attention to how wastewater data are shared, interpreted, and presented [108]. Together, these reports emphasize that mature WBS depends on system design, data integration, and reporting infrastructure in addition to analytical methods. This is consistent with the discussion in the section on methodological complexities, where transferability depends on cross-site differences in data generation and surveillance context.

#### Interoperable data and analytical reporting

Interoperable data structures provide a concrete area for analytical standardization. The PHES-ODM data model introduced in the section on data harmonization and normalization provides an early implementation, specifying how sites, samples, measurements, methods, and contextual metadata are represented to support more consistent combination of datasets across programmes [80]. An equivalent convention for analytical reporting, however, has not yet been established: no widely adopted standard exists for reporting the model family or analytical approach used, the validation design, the treatment of censored measurements, or the baseline against which performance was assessed. As discussed in the section on methodological complexities, these details are often reported in insufficient depth to support clear interpretation of published results. Extending harmonization from measurement to analytical reporting would improve the comparability and interpretation of published models.

#### Cross-disciplinary co-design

Such interoperability and reporting frameworks should be co-designed by data scientists, microbiologists, engineers, wastewater utilities, epidemiologists, and public-health agencies. The interdisciplinary coordination barriers described in the section on real-world implementation indicate that their development will require deliberate cross-disciplinary coordination, including shared forums, agreements, and translation between technical and public-health domains.

### Multimodal data fusion and integrated surveillance

Multimodal fusion can occur both analytically, through the integration of heterogeneous data streams, and operationally, through complementary sampling that broadens population and infrastructure coverage.

#### Integration of epidemiological, mobility, and sequencing data

Multimodal fusion can integrate wastewater signals with the epidemiological and contextual data needed for their interpretation. Potential inputs include wastewater measurements, clinical and syndromic indicators, anonymized mobility data, environmental and operational variables, and amplicon or metagenomic sequencing data from both wastewater and clinical samples [60,120,121]. Existing studies show that combining selected data streams can add information. Schenk et al. compared four SARIMAX specifications for predicting SARS-CoV-2 cases in Liechtenstein and two Austrian regions – active cases alone, active cases with wastewater data, active cases with mobility data, and a combined model incorporating restriction policies – and found that adding mobility data and restriction information improved predictive precision beyond what wastewater measurements alone provided [57]. At the spatial scale, Tierney et al. integrated targeted amplicon and bulk metatranscriptomic sequencing across 2,238 samples from geospatially distinct areas of Miami-Dade County, tracked SARS-CoV-2 variants with close correspondence to PCR results from university students and hospital patients, and showed that bacterial content at sampling points serving small populations carried information on host-derived microorganisms associated with human health and diet [69]. Future spatio-temporal models could integrate pathogen circulation, human behaviour, environmental conditions, and local infrastructure, while being evaluated for interpretability and external validity under the designs set out in the section on methodological complexities [61,69]. Where data-sharing restrictions rather than model structure are the main constraint, federated approaches remain a prospective route to cross-site modelling without exchanging raw data, as noted in the section on methodological complexities [103].

#### Complementary sampling for population representativeness

Fusion need not begin at the modelling stage. A complementary strategy is to design sampling protocols that capture biological information missing from the sewer signal, addressing at the point of collection the representativeness gaps described in the section on data quality and availability. In long-term care facilities for people with intellectual disabilities, a study protocol has been registered that combines passive sampling of facility sewers with culture of stool obtained from diaper material, in order to estimate the burden of extended-spectrum beta-lactamase- and carbapenemase-producing Enterobacterales among residents for whom rectal swabbing is considered too burdensome [122]. The protocol illustrates how collection design can be used to address representativeness rather than relying on computational correction alone. Its stated limitations also define what facility-level sampling cannot resolve: wastewater measurements cannot identify individual carriers or distinguish the contributions of residents from those of staff and visitors. Results are not yet available, and the approach remains to be evaluated.

#### Multimodal surveillance beyond centralized sewer networks

The same principle extends to settings with partial or absent sewer coverage. The complementary matrices described in the section on real-world implementation can capture signals from populations that are poorly represented or absent from sewer-derived sampling, and passive samplers can integrate signals over time where automated composite sampling is not feasible. Combining these matrices with sewer-derived data creates a multimodal fusion problem in which the streams differ in catchment definition, sampling frequency, and recovery efficiency. Models designed for this setting would need to represent those differences explicitly, through site-level random effects or matrix-specific measurement models, rather than treating all samples as exchangeable observations of a single underlying concentration. Such additional data streams could improve population representativeness and provide more informative inputs for model calibration and public-health interpretation.

### Mechanistic modelling and uncertainty with digital twins

A coupled wastewater digital twin requires an epidemiological and infrastructure model, coherent treatment of uncertainty across the two components, and explicit validation before scenario-based use.

#### Epidemiological and infrastructure components

A key future direction is to move beyond purely correlational forecasting toward mechanistic simulation and scenario-based decision support. Digital twins – dynamic digital representations of real-world systems – can in principle integrate wastewater measurements, epidemiological models, mobility patterns, sewer hydraulics, and ML analytics to evaluate how observed wastewater signals relate to underlying infection dynamics. The Catalan digital twin introduced in the section on predictive modelling and forecasting illustrates the epidemiological component of such a coupling: an extended SEIRD simulation of SARS-CoV-2 transmission, continuously validated against the regional sewage surveillance network, in which sewage trends are used to detect discrepancies between simulated and observed dynamics and thereby to reassess the model’s assumptions [11]. The authors are explicit that their aim is not to estimate case numbers from viral loads but to use the wastewater signal as an independent check on a mechanistic model whose clinical inputs had become unreliable as testing policy narrowed. The study reports agreement between simulated and observed trends rather than a formal assessment of predictive uncertainty.

The infrastructure component comes from a different literature. Digital twins for sewer networks and wastewater treatment systems combine real-time sensing, hydraulic models, and data-driven analytics to support simulation, forecasting, and operational control of wastewater infrastructure [84]. Lumley et al. implemented such a twin on the Future City Flow platform for Gothenburg’s regional sewage system, integrating modelling, visualization, forecasting, and model-based predictive control that recommends operational setpoints during storms [83]. Neither of these systems was developed for epidemiological surveillance, and neither has been coupled to a transmission model; they are cited here as the infrastructure layer such a coupling would require rather than as evidence that it has been achieved. They also indicate where uncertainty in that layer originates: the Gothenburg authors identify high-quality online catchment data and, above all, high-quality rainfall forecasts as key requirements, particularly over long prediction horizons [83]. Storm periods are therefore a particularly important source of hydraulic uncertainty.

#### Uncertainty propagation and calibration

Coupling the two components raises a question that neither addresses on its own: how uncertainty behaves once they are joined. In a coupled framework, uncertainty propagates along a chain running from sampling and analytical variability, through the estimated concentration, through the hydraulic and in-sewer decay parameters used to convert that concentration into a load, through the inferred number of infections, to the epidemiological state and finally the predicted indicator (Figure 5). These contributions do not simply add. Because load is the product of concentration and flow, uncertainty in these two quantities propagates through a multiplicative step; under the usual small-error and independence approximation, their relative uncertainties combine approximately in quadrature. Uncertainty is therefore likely to be greatest in two operationally important regimes: during storm events, when flow is high and dependent on rainfall forecasts that are themselves uncertain [83], and during low-prevalence periods, when a large proportion of concentration measurements is censored (see the section on data quality and availability). A coupled framework that reports a single uncertainty band without distinguishing these regimes may obscure or understate regime-specific uncertainty.

**Figure 5.**
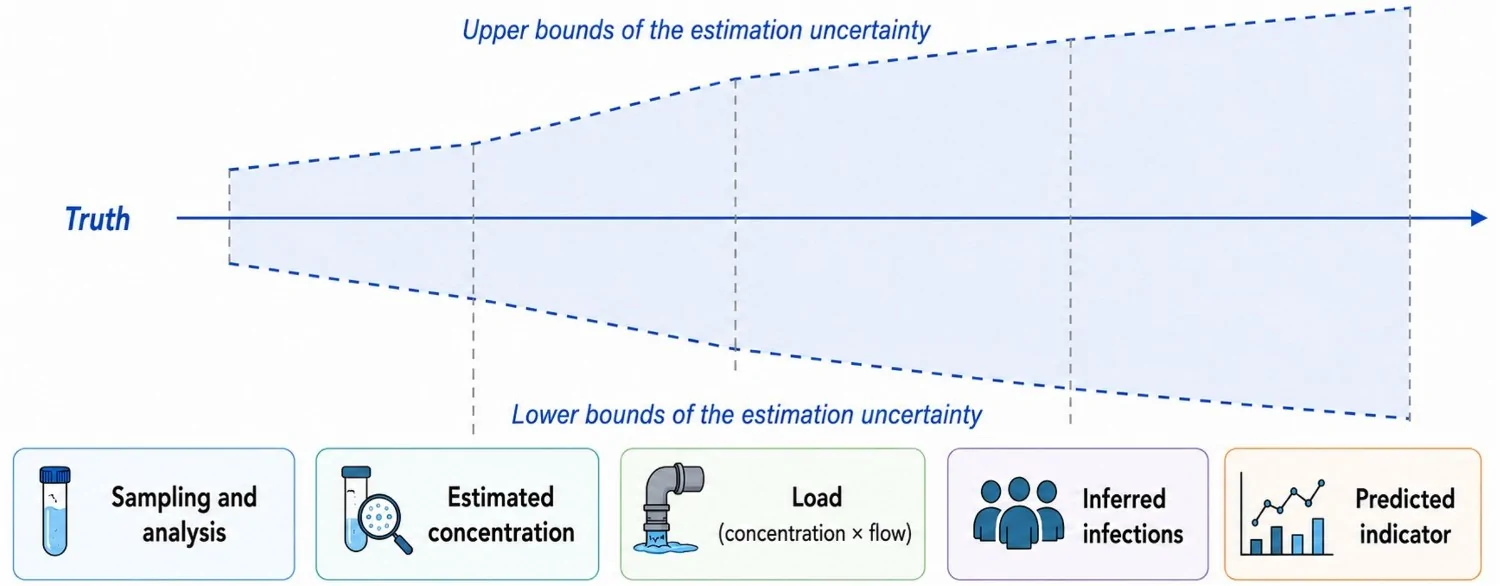
Growth of uncertainty along a coupled wastewater digital-twin chain. The horizontal line represents the true value at each stage of the chain, and the envelope around it represents the uncertainty attached to that estimate; its vertical extent at any point is the magnitude of uncertainty at that stage. Each stage introduces further uncertainty, which is carried forward into all subsequent stages, so the envelope widens from left to right. The widening is gradual across the additive steps but may become particularly pronounced at the conversion of concentration into load, because load is the product of concentration and flow and, under a small-error and independence approximation, the relative uncertainties of the two factors combine approximately in quadrature rather than adding directly. This expansion may be particularly large during storm events, when flow is high and poorly constrained, and during low-prevalence periods, when a large proportion of concentration measurements is censored.

A second issue is that the two components may quantify different objects. A probabilistic data-driven model may report a predictive interval for a future observation, whereas a mechanistic model may report uncertainty around a latent state or parameter. These intervals are not directly interchangeable because they can represent different sources of uncertainty. Simply propagating one through the other does not guarantee that the nominal interval level will match empirical coverage. Two routes can address this. The first is joint inference, in which the mechanistic structure and the data-driven components are estimated within a single probabilistic model so that a coherent posterior predictive distribution is obtained directly; the semi-mechanistic Bayesian framework discussed in the section on predictive modelling and forecasting illustrates this approach [59]. The second is post hoc verification: whatever the internal construction, the coupled output should be assessed under the established paradigm for probabilistic forecasts, which is to maximize sharpness subject to calibration, using diagnostics such as the probability integral transform histogram, marginal calibration plots, and proper scoring rules [123]. Current reporting practice in wastewater-informed forecasting rarely distinguishes these forms of uncertainty.

#### Validation criteria for scenario testing

Such hybrid frameworks could provide virtual environments for testing surveillance strategies, sampling designs, and resource-allocation scenarios before real-world deployment. Their suitability for scenario testing should be assessed against explicit criteria. Three are particularly relevant. First, a twin intended for strategy testing should reproduce known failure modes of the real system, such as dilution during storm events or the shift in catchment population during holiday periods; a model that reproduces only routine behaviour provides limited evidence about the conditions under which a sampling design would fail. Second, quantities that the twin infers but does not observe, such as the number of infections implied by a measured load, should be checked against an independent source. The longitudinal serological study conducted alongside wastewater surveillance in Alberta long-term care facilities, in which neutralising and anti-nucleocapsid antibody status was measured in residents across four periods of the vaccination programme, illustrates the kind of external reference this requires [78], since internal consistency between the hydraulic and epidemiological components does not establish that an inferred quantity is correct. Third, any scenario comparison should report how its conclusion changes across the plausible range of calibrated parameters; where the ordering of two strategies reverses within that range, the result should be treated as sensitivity-dependent rather than as robust decision support. Applied in this way, digital twins can make the assumptions underlying causal claims explicit and testable, rather than being treated as a direct route to causal inference from wastewater data.

### Transparent, interpretable, and actionable analytics

Transparent and actionable ML-enabled WBS requires model explanation, operational decision rules, explicit communication of uncertainty, and deployment designs suited to local capacity.

#### Explainability and model validation

Future adoption of ML-enabled WBS will require analytics that are transparent and interpretable and outputs that are operationally actionable within ethically governed surveillance systems. One technical response to model opacity is to pair predictive models with explainable-AI (XAI) methods and present the resulting explanations alongside numerical predictions. These methods can help identify which inputs most influence a model’s prediction, but they do not establish whether the prediction is correct or quantify its uncertainty. The SHAP analysis discussed in the section on predictive modelling and forecasting provides the only WBS-specific demonstration identified here, in which feature attribution showed that catchment population and the N gene indicator were the most influential features in an extreme-gradient-boosting model of case counts [55]. Outside this field, a review of 27 studies on explaining machine-learning models for digital health data concluded that explanation methods require further development and validation before they can be relied upon in precision healthcare [85]; the same caution applies to wastewater analytics, where the evidence base is considerably thinner. Model explanations should therefore complement, rather than replace, validation and uncertainty assessment.

#### Operational translation into decision support

Model outputs must also be translated into tools that support rapid decisions, such as thresholds, alerts, uncertainty displays, and dashboards (see the section on real-world implementation). Four operational functions are particularly relevant.

- Dynamic thresholding. Static thresholds are vulnerable to environmental change, site-specific variation, and seasonal fluctuation: a concentration associated with surge conditions in one catchment or season may be unremarkable in another, so a fixed cut-off can generate both false alarms and missed signals. Data-derived or site-specific thresholds provide one alternative. In the study discussed in the section on anomaly and fluctuation detection, a clustering model using wastewater, mobility, and clinical data distinguished surge weeks from inter-surge periods with 87.9% accuracy [50]. This demonstrates data-driven risk stratification, but not prospective deployment of an adaptive alert threshold. A clinically anchored threshold requires an outcome series against which the cut-off can be calibrated. Where such data are unavailable, site-specific historical distributions can provide anomaly thresholds, although their public-health meaning still requires validation.
- Early-warning alerts. Supervised time-series models can forecast a wastewater-derived or epidemiological indicator and support an alert when the forecast crosses a pre-specified risk boundary. Long short-term memory networks (L3) have been applied to multi-community outbreak prediction in WBS [52]; we identified no WBS evaluation of gated recurrent units for this purpose. The operational value of such alerts depends on lead time and false-alarm rate, not on point accuracy alone, and these metrics should be reported at a stated forecast horizon (see the section on methodological complexities).
- Communication of uncertainty. Dashboards can present prediction intervals rather than point estimates alone. One general method is quantile regression forests, which estimate conditional quantiles directly from a fitted ensemble [124]. Because the resulting interval width can vary with the covariates, this approach offers a way to represent predictive uncertainty that changes with the modelled conditions. This flexibility may be useful in WBS, where predictive uncertainty can vary across low-prevalence and storm conditions (see the sections on data quality and availability and on mechanistic modelling and digital twins). The interval's coverage must be verified against observation, and where intervals from data-driven and mechanistic components are displayed together, their differing meaning must be stated (see the section on mechanistic modelling and digital twins).
- Deployment under resource constraints. Under resource constraints, simpler models may be preferable when they meet the required operational objective. This matters where computational capacity, technical staff, and network connectivity are limited (see the section on real-world implementation). Local or edge deployment may reduce dependence on continuous network connectivity and external cloud services and can help keep surveillance data within the jurisdiction in which they are generated. As argued in the section on anomaly and fluctuation detection, a more complex model should be adopted only when it improves a pre-specified operational metric against both a naive baseline and the best available simpler alternative.

Beyond technical transparency, ML-enabled WBS will require proactive governance frameworks that define permissible data uses, assign accountability, prevent function creep, and ensure that wastewater-derived intelligence is managed responsibly as surveillance systems scale (see the section on real-world implementation). Operational deployment remains prospective: in the evidence identified in this review, none of these four ML-enabled functions has been prospectively evaluated as part of a routine surveillance programme. Multi-setting validation is therefore needed before they are relied upon for public-health action.

### Beyond pathogen monitoring

The scope of ML-enabled WBS is extending beyond pathogen monitoring into broader population-health surveillance, notably substance use and antimicrobial resistance (AMR). These applications pose different analytical problems: substance-use surveillance requires harmonization and spatiotemporal integration across analytes and locations, whereas AMR surveillance generates high-dimensional molecular data requiring sequence annotation and interpretation.

#### Substance-use surveillance and data integration

Substance-use monitoring is one of the most established non-infectious applications of WBE. Laboratories quantify drug residues and human metabolites in municipal wastewater, aggregate the signals by catchment and sampling period, and infer city-level temporal and geographical patterns of use. A 2023 survey coordinated by SCORE and the European Union Drugs Agency found an established European research and stakeholder infrastructure, with two-thirds of participating countries already using wastewater data to inform drug policy [71]. The same survey identified limitations in generating national estimates from a limited number of sampled cities, sampling frequency for policy use in some countries, and cross-indicator analysis [71]. These limitations echo the problems of representativeness, temporal resolution, and data integration discussed in the section on challenges.

#### AMR and high-dimensional resistome analysis

AMR surveillance generates complex population-level molecular data of a different kind. A national programme sampling 47 treatment plants across Wales compared high-throughput qPCR against 73 resistance genes with metagenomic sequencing, and found the two approaches complementary: metagenomics detected 545 genes and provided the bacterial host context needed for risk assessment, whereas HT-qPCR quantified all targeted genes including clinically relevant ones present at low abundance, and both recovered the same spatiotemporal separation between hospital and municipal resistomes [67]. At the analytical level, deep-learning-based ARG annotation provides a relevant methodological tool, although the evidence cited here comes from bioinformatics rather than from an ML model deployed in WBS [68]. ML could contribute to AMR surveillance through sequence annotation, spatial pattern analysis, and interpretation of high-dimensional resistome profiles using approaches such as those reviewed in the section on visualization and interpretation of high-dimensional data.

#### Endogenous biomarkers and broader health signals

Wastewater can also capture endogenous biomarkers beyond infectious and resistance-related targets. Endogenous stress hormones have been quantified in wastewater from a university campus of approximately 26,000 people, with reproducible weekly cycles in cortisol and cortisone excretion, significantly higher levels on weekdays than at weekends, and elevated levels during the first two months of each semester [70]; expert commentary describes wastewater as carrying endogenous and exogenous metabolites reflecting lifestyle, diet, exposure, and health status alongside pathogens [125]. These sources establish the feasibility of measuring broader population-level biomarkers, not the performance of ML models using them. Combining such biomarkers with chemical, environmental, and contextual measurements could nevertheless create multimodal datasets for future ML analysis.

#### Governance and public acceptability

Technical feasibility does not by itself establish that an application is acceptable or appropriate for surveillance. The public survey discussed in the section on real-world implementation found support for wastewater surveillance of disease at 91% but of lifestyle behaviours at 35%, of healthy eating at 37%, and of mental illness at 46%, and found support declining as the geographic scale became smaller and less anonymous [117]. These findings show that acceptability depends on both the surveillance target and spatial resolution. Expansion toward behavioural or lifestyle monitoring should therefore be preceded by explicit governance and community-engagement frameworks rather than introduced solely because the measurements are technically possible.

Together, these examples broaden the potential scope of ML-enabled WBS beyond pathogen trend monitoring, but the maturity of the evidence differs substantially among applications. Substance-use WBE is operationally established, AMR produces data well suited to high-dimensional analysis but has limited demonstrated ML deployment, and endogenous-biomarker studies establish measurement feasibility rather than ML-enabled decision support. Broader population-health surveillance should therefore develop in step with both analytical evidence and public acceptability.

## Summary

### Analytical framework and strength of evidence

Wastewater-based surveillance has become an important population-level tool for monitoring infectious disease dynamics, but wastewater measurements are not directly equivalent to public-health indicators. Their interpretation requires analytical workflows that can distinguish true epidemiological signals from technical, environmental, and population-related noise. This review organized the analytical methods used in WBS into four layers – classical statistical models, conventional machine learning, deep learning, and hybrid or mechanistically informed approaches – and applied that classification consistently across applications, challenges, and future directions. Classical statistical models remain central to trend description, correlation and lag–lead analysis, signal processing, and early-warning interpretation, and account for most of the demonstrated results reviewed here; machine learning adds capacity for heterogeneous, high-dimensional, nonlinear, and multimodal data, and demonstrated gains from ML have been concentrated mainly in multivariable settings where clinical and contextual covariates are available.

Current ML applications in WBS span predictive modelling and forecasting, anomaly and fluctuation detection, data harmonization and normalization, high-dimensional interpretation, and integrated decision support. The strength of the evidence supporting them is markedly uneven (Table 2). Quantitative modelling evidence is strongly concentrated in SARS-CoV-2, for which hospitalization and case forecasting, outbreak-risk classification, and trend-deviation detection have all been reported. For influenza viruses and respiratory syncytial virus, published work remains correlational or descriptive; for enteric and hepatitis viruses, mpox virus, poliovirus, bacterial and protozoan pathogens, and antimicrobial resistance determinants, machine-learning applications are at present prospective rather than demonstrated. Conclusions about the predictive and early-warning performance of ML in WBS should therefore be interpreted primarily in the context of SARS-CoV-2 surveillance under unusually favourable data conditions, and generalization to other pathogens remains an open question. Even within SARS-CoV-2 surveillance, reported performance is strongly context-dependent: forecast accuracy depends strongly on sampling frequency and training-set size [58], and adding wastewater data improved or degraded forecasts substantially at particular locations and dates while leaving overall performance essentially unchanged [59].

### Conditions for reliable application

Practical reliability is constrained by a further set of factors that operate upstream of any model: data incompleteness and censoring, sampling variability, inconsistent laboratory and reporting practices, limited external validation under time- and space-aware designs, model opacity, operational cost, and governance requirements. These constraints affect model families differently. Many L1 methods can be fitted to the wastewater series itself, whereas supervised L2 and L3 models generally require paired outcomes and larger training sets; L4 approaches additionally require mechanistic or system-level information. Computational and maintenance demands also vary with model complexity and deployment context. Greater model complexity should therefore be justified by improvement in a pre-specified operational metric against both a naive baseline and the best available simpler alternative. ML is most appropriately used as decision support within broader public-health surveillance and expert interpretation, rather than as an autonomous decision system.

### Future development and scope

Future WBS systems may develop from pathogen-specific monitoring platforms into integrated population-health intelligence systems supporting outbreak preparedness, pandemic response, and One Health surveillance. Realizing this will require closing the gaps identified in Table 2, in particular the scarcity of validated ML models for targets other than SARS-CoV-2 and for settings not served by centralized sewer networks. Progress will depend on standardized collaborative frameworks, harmonized data infrastructures, multimodal data fusion, mechanistic and digital-twin modelling with explicit uncertainty assessment, transparent and explainable analytics, and governance mechanisms that define responsible data use and accountability. The scope of ML-enabled WBS could also extend beyond pathogen monitoring toward antimicrobial resistance, substance use, and other population-health biomarkers. Public acceptability is substantially lower for behavioural and lifestyle targets and at smaller geographic scales (see the section on real-world implementation), so such expansion will need to reflect public acceptability as well as analytical feasibility. Under these conditions, ML can help move WBS from retrospective signal reporting toward proactive, interpretable, and scalable public-health surveillance.

## Supplementary Material

Song WWS Supplementary materials.docx

## Declaration

AI-assisted tools were used only for ancillary editorial and organizational support, including language polishing, figure and table polishing, literature organization, and reference indexing. Literature selection, evidence assessment, scientific interpretation, and all conclusions were reviewed and verified by the authors.

## References

[1] Larsen DA, Wigginton KR. Tracking COVID-19 with wastewater. Nat Biotechnol. 2020 Oct;38(10):1151–1153. doi:10.1038/s41587-020-0690-1 PMC7505213 32958959

[2] Polo D, Quintela-Baluja M, Corbishley A, et al. Making waves: wastewater-based epidemiology for COVID-19–approaches and challenges for surveillance and prediction. Water Res. 2020 Nov 1;186:116404. doi:10.1016/j.watres.2020.116404 PMC7480445 32942178

[3] World Health Organization. Environmental surveillance for SARS-CoV-2 to complement other public health surveillance. World Health Organization; 2023 Sep 22. Available from: https://www.who.int/publications/i/item/9789240080638

[4] Bowes DA, Driver EM, Choi PM, et al. Wastewater-based epidemiology to assess environmentally influenced disease. J Expo Sci Environ Epidemiol. 2024 May;34(3):387–388. doi:10.1038/s41370-024-00683-w PMC11912583 38760533

[5] Hovi T, Shulman LM, Van Der Avoort H, et al. Role of environmental poliovirus surveillance in global polio eradication and beyond. Epidemiol Infect. 2012 Jan;140(1):1–13. doi:10.1017/S095026881000316X 21849095

[6] Hill M, Pollard AJ. Detection of poliovirus in London highlights the value of sewage surveillance. Lancet. 2022 Oct 29;400(10362):1491–1492. doi:10.1016/S0140-6736(22)01885-2 36243023

[7] Klapsa D, Wilton T, Zealand A, et al. Sustained detection of type 2 poliovirus in London sewage between February and July, 2022, by enhanced environmental surveillance. Lancet. 2022;400(10362):1531–1538. doi:10.1016/S0140-6736(22)01804-9 PMC9627700 36243024

[8] Medema G, Heijnen L, Elsinga G, et al. Presence of SARS-coronavirus-2 RNA in sewage and correlation with reported COVID-19 prevalence in the early stage of the epidemic in the Netherlands. Environ Sci Technol Lett. 2020;7(7):511–516. doi:10.1021/acs.estlett.0c00357 37566285

[9] Peccia J, Zulli A, Brackney DE, et al. Measurement of SARS-CoV-2 RNA in wastewater tracks community infection dynamics. Nat Biotechnol. 2020 Oct;38(10):1164–1167. doi:10.1038/s41587-020-0684-z PMC8325066 32948856

[10] Hamouda M, Mustafa F, Maraqa M, et al. Wastewater surveillance for SARS-CoV-2: lessons learnt from recent studies to define future applications. Sci Total Environ. 2021 Mar 10;759:143493. doi:10.1016/j.scitotenv.2020.143493 PMC7648500 33190883

[11] Fonseca i Casas P, Garcia i Subirana J, Corominas L, et al. Applying a digital twin and wastewater analysis for robust validation of COVID-19 pandemic forecasts: insights from Catalonia. J Water Health. 2024 Mar 1;22(3):584–600. doi:10.2166/wh.2024.345 38557573

[12] European Commission (HERA). The European Commission lays the foundations for a global system for wastewater surveillance for public health; 2023 Nov 14. Available from: https://health.ec.europa.eu/latest-updates/european-commission-lays-foundations-global-system-wastewater-surveillance-public-health-2023-11-14_en

[13] CDC (Centers for Disease Control and Prevention). Wastewater surveillance data reporting and analytics (NWSS); 2024 Jul 31. Available from: https://archive.cdc.gov/www_cdc_gov/nwss/reporting.html

[14] CDC (Centers for Disease Control and Prevention). About wastewater data (NWSS); 2025 Aug 12. Available from: https://www.cdc.gov/wastewater/about-data/index.html

[15] European Commission. Launching the EU Wastewater Surveillance Dashboard; 2025 Jan 29. Available from: https://health.ec.europa.eu/latest-updates/launching-eu-wastewater-surveillance-dashboard-2025-01-29_en

[16] Gibas C, Lambirth K, Mittal N, et al. Implementing building-level SARS-CoV-2 wastewater surveillance on a university campus. Sci Total Environ. 2021 Aug 15;782:146749. doi:10.1016/j.scitotenv.2021.146749 PMC8007530 33838367

[17] Li X, Liu H, Gao L, et al. Wastewater-based epidemiology predicts COVID-19-induced weekly new hospital admissions in over 150 USA counties. Nat Commun. 2023 Jul 28;14(1):4548. doi:10.1038/s41467-023-40305-x PMC10382499 37507407

[18] Schenk H, Heidinger P, Insam H, et al. Prediction of hospitalisations based on wastewater-based SARS-CoV-2 epidemiology. Sci Total Environ. 2023 May 15;873:162149. doi:10.1016/j.scitotenv.2023.162149 PMC9911153 36773921

[19] Daughton CG. Wastewater surveillance for population-wide Covid-19: The present and future. Sci Total Environ. 2020 Sep 20;736:139631. doi:10.1016/j.scitotenv.2020.139631 PMC7245244 32474280

[20] Sims N, Kasprzyk-Hordern B. Future perspectives of wastewater-based epidemiology: monitoring infectious disease spread and resistance to the community level. Environ Int. 2020 Jun 1;139:105689. doi:10.1016/j.envint.2020.105689 PMC7128895 32283358

[21] Malla B, Shrestha S, Sthapit N, et al. Beyond COVID-19: wastewater-based epidemiology for multipathogen surveillance and normalization strategies. Sci Total Environ. 2024 Oct 10;946:174419. doi:10.1016/j.scitotenv.2024.174419 38960169

[22] Parkins MD, Lee BE, Acosta N, et al. Wastewater-based surveillance as a tool for public health action: SARS-CoV-2 and beyond. Clin Microbiol Rev. 2024 Mar 14;37(1):e00103–22. doi:10.1128/cmr.00103-22 PMC10938902 38095438

[23] Singh S, Ahmed AI, Almansoori S, et al. A narrative review of wastewater surveillance: pathogens of concern, applications, detection methods, and challenges. Front Public Health. 2024 Jul 30;12:1445961. doi:10.3389/fpubh.2024.1445961 PMC11319304 39139672

[24] Louis S, Mark-Carew M, Biggerstaff M, et al. Wastewater surveillance for influenza A virus and H5 subtype concurrent with the highly pathogenic avian influenza A(H5N1) virus outbreak in cattle and poultry and associated human cases – United States, May 12–July 13, 2024. MMWR Morb Mortal Wkly Rep. 2024 Sep 19;73(37):804–809. doi:10.15585/mmwr.mm7337a1 PMC11412440 39298357

[25] Zulli A, Varkila MR, Parsonnet J, et al. Observations of respiratory syncytial virus (RSV) nucleic acids in wastewater solids across the United States in the 2022–2023 season: relationships with RSV infection positivity and hospitalization rates. ACS ES&T Water. 2024 Feb 29;4(4):1657–1667. doi:10.1021/acsestwater.3c00725 PMC11019535 38633368

[26] Erster O, Bar-Or I, Levy V, et al. Monitoring of enterovirus D68 outbreak in Israel by a parallel clinical and wastewater based surveillance. Viruses. 2022 May 9;14(5):1010. doi:10.3390/v14051010 PMC9144596 35632752

[27] Darling A, Davis BC, Byrne T, et al. Comparative assessment of wastewater-based surveillance normalization methods to improve pathogen monitoring in rural sewersheds. Environ Sci Technol. 2025 May 27;59(22):11095–11107. doi:10.1021/acs.est.4c14485 PMC12164260 40420392

[28] Boehm AB, Shelden B, Duong D, et al. A retrospective longitudinal study of adenovirus group F, norovirus GI and GII, rotavirus, and enterovirus nucleic acids in wastewater solids at two wastewater treatment plants: solid-liquid partitioning and relation to clinical testing data. mSphere. 2024 Mar 26;9(3):e00736–23. doi:10.1128/msphere.00736-23 PMC10964402 38411118

[29] Veneri C, Bonanno Ferraro G, Congiu D, et al. Wastewater-based surveillance of human adenoviruses in Italy: quantification by digital PCR and molecular typing via nanopore amplicon sequencing. Viruses. 2025 May 30;17(6):791. doi:10.3390/v17060791 PMC12197328 40573383

[30] Bowes DA, Henke KB, Driver EM, et al. Enhanced detection of mpox virus in wastewater using a pre-amplification approach: A pilot study informing population-level monitoring of low-titer pathogens. Sci Total Environ. 2023 Dec 10;903:166230. doi:10.1016/j.scitotenv.2023.166230 PMC10592092 37574063

[31] Schenk H, Rauch W, Zulli A, et al. SARS-CoV-2 surveillance in US wastewater: leading indicators and data variability analysis in 2023–2024. PLoS One. 2024 Nov 18;19(11):e0313927. doi:10.1371/journal.pone.0313927 PMC11573121 39556598

[32] Ibrahim C, Hammami S, Pothier P, et al. The performance of biological and tertiary wastewater treatment procedures for rotaviruses A removal. Environ Sci Pollut Res. 2020 Feb;27(6):5718–5729. doi:10.1007/s11356-019-05487-2 PMC7223008 31177419

[33] Zulli A, Chan EM, Boehm AB. Detection of Hepatovirus A (HAV) in wastewater indicates widespread national distribution and association with socioeconomic indicators of vulnerability. mSphere. 2024 Nov 21;9(11):e00645–24. doi:10.1128/msphere.00645-24 PMC11580403 39475316

[34] Rau F, Elsner C, Meister TL, et al. Monitoring of hepatitis E virus in wastewater can identify clinically relevant variants. Liver Int. 2024 Mar;44(3):637–643. doi:10.1111/liv.15842 38291853

[35] Kim YT, Lee K, Lee H, et al. Development of a wastewater based infectious disease surveillance research system in South Korea. Sci Rep. 2024 Oct 19;14(1):24544. doi:10.1038/s41598-024-76614-4 PMC11490628 39427054

[36] Yang K, Pagaling E, Yan T. Estimating the prevalence of potential enteropathogenic escherichia coli and intimin gene diversity in a human community by monitoring sanitary sewage. Appl Environ Microbiol. 2014 Jan 1;80(1):119–127. doi:10.1128/AEM.02747-13 PMC3910997 24141131

[37] Hamilton KA, Waso M, Reyneke B, et al. Cryptosporidium and Giardia in wastewater and surface water environments. J Environ Qual. 2018 Sep;47(5):1006–1023. doi:10.2134/jeq2018.04.0132 30272766

[38] Ajonina C, Buzie C, Möller J, et al. The detection of Entamoeba histolytica and Toxoplasma gondii in wastewater. J Toxicol Environ Health, Part A. 2018 Feb 1;81(1-3):1–5. doi:10.1080/15287394.2017.1392399 29173133

[39] Ahmed W, Smith WJ, Metcalfe S, et al. Comparison of RT-qPCR and RT-dPCR platforms for the trace detection of SARS-CoV-2 RNA in wastewater. ACS ES&T Water. 2022 Jan 28;2(11):1871–1880. doi:10.1021/acsestwater.1c00387 PMC8848507 36380768

[40] Chai X, Liu S, Liu C, et al. Surveillance of SARS-CoV-2 in wastewater by quantitative PCR and digital PCR: a case study in Shijiazhuang city, Hebei province, China. Emerging Microbes Infect. 2024 Dec 31;13(1):2324502. doi:10.1080/22221751.2024.2324502 PMC10930138 38465692

[41] Chik AHS, Adam C, Ho JJY, et al. One for all and all for one health: harmonizing SARS-CoV-2 wastewater surveillance results across a laboratory network. Sci Total Environ. 2025 Oct 15;999:180200. doi:10.1016/j.scitotenv.2025.180200 40857943

[42] Maal-Bared R, Qiu Y, Li Q, et al. Does normalization of SARS-CoV-2 concentrations by Pepper Mild Mottle Virus improve correlations and lead time between wastewater surveillance and clinical data in Alberta (Canada): comparing twelve SARS-CoV-2 normalization approaches. Sci Total Environ. 2023 Jan 15;856(Pt 1):158964. doi:10.1016/j.scitotenv.2022.158964 PMC9508694 36167131

[43] Holm RH, Nagarkar M, Yeager RA, et al. Surveillance of RNase P, PMMoV, and CrAssphage in wastewater as indicators of human fecal concentration across urban sewer neighborhoods, Kentucky. FEMS Microbes. 2022;3:xtac003. doi:10.1093/femsmc/xtac003 PMC10117713 37228897

[44] Safford H, Zuniga-Montanez RE, Kim M, et al. Wastewater-based epidemiology for COVID-19: handling qPCR nondetects and comparing spatially granular wastewater and clinical data trends. ACS ES&T Water. 2022 Jul 29;2(11):2114–2124. doi:10.1021/acsestwater.2c00053 37552742

[45] Lewis-Borrell L, Irving J, Lilley CJ, et al. Robust smoothing of left-censored time series data with a dynamic linear model to infer SARS-CoV-2 RNA concentrations in wastewater. AIMS Math. 2023;8(7):16790–16824. doi:10.3934/math.2023859

[46] Wade MJ, Jacomo AL, Armenise E, et al. Understanding and managing uncertainty and variability for wastewater monitoring beyond the pandemic: lessons learned from the United Kingdom national COVID-19 surveillance programmes. J Hazard Mater. 2022 Feb 15;424:127456. doi:10.1016/j.jhazmat.2021.127456 PMC8498793 34655869

[47] Ensor KB, Schedler JC, Sun T, et al. Online trend estimation and detection of trend deviations in sub-sewershed time series of SARS-CoV-2 RNA measured in wastewater. Sci Rep. 2024 Mar 6;14(1):5575. doi:10.1038/s41598-024-56175-2 PMC10918082 38448481

[48] Manuel DG, Saran G, Lee I, et al. Wastewater-based surveillance of SARS-CoV-2: short-term projection (forecasting), smoothing and outlier identification using Bayesian smoothing. Sci Total Environ. 2024;949:174937. doi:10.1016/j.scitotenv.2024.174937 39067598

[49] Saravia CJ, Pütz P, Wurzbacher C, et al. Wastewater-based epidemiology: deriving a SARS-CoV-2 data validation method to assess data quality and to improve trend recognition. Front Public Health. 2024 Dec;12:1497100. doi:10.3389/fpubh.2024.1497100 PMC11674844 39735750

[50] Armenta-Castro A, de la Rosa O, Aguayo-Acosta A, et al. Interpretation of COVID-19 epidemiological trends in Mexico through wastewater surveillance using simple machine learning algorithms for rapid decision-making. Viruses. 2025 Jan 15;17(1):109. doi:10.3390/v17010109 PMC11768489 39861898

[51] Lai M, Cao Y, Wulff SS, et al. A time series based machine learning strategy for wastewater-based forecasting and nowcasting of COVID-19 dynamics. Sci Total Environ. 2023 Nov 1;897:165105. doi:10.1016/j.scitotenv.2023.165105 37392891

[52] Ai Y, He F, Lancaster E, et al. Application of machine learning for multi-community COVID-19 outbreak predictions with wastewater surveillance. PLoS One. 2022;17(11):e0277154. doi:10.1371/journal.pone.0277154 PMC9648834 36355921

[53] Dejus B, Cacivkins P, Gudra D, et al. Wastewater-based prediction of COVID-19 cases using a random forest algorithm with strain prevalence data: a case study of five municipalities in Latvia. Sci Total Environ. 2023 Sep 15;891:164519. doi:10.1016/j.scitotenv.2023.164519 PMC10229444 37268136

[54] Tabassum N, Chowdhury MM, McMahan CS, et al. Granular insights: a wastewater-based machine learning approach for localized COVID-19 hospitalization forecasting. Epidemics. 2026 Mar 24;55:100907. doi:10.1016/j.epidem.2026.100907 PMC13746233 41932030

[55] Ribalta M, Pueyo-Ros J, Corominas L, et al. Explainable machine learning models for predicting COVID-19 cases in Catalonia based on wastewater monitoring data. In: Sanz I, Ros R, Nin J, editors. Artificial intelligence research and development. Amsterdam: IOS Press; 2023. p. 262–266. doi:10.3233/FAIA230691

[56] Tang S, Cao Y. A phenomenological neural network powered by the national wastewater surveillance system for estimation of silent COVID-19 infections. Sci Total Environ. 2023;902:166024. doi:10.1016/j.scitotenv.2023.166024 37541490

[57] Schenk H, Arabzadeh R, Dabiri S, et al. Integrating wastewater-based epidemiology and mobility data to predict SARS-CoV-2 cases. Environments. 2024 May 12;11(5):100. doi:10.3390/environments11050100

[58] Vaughan L, Zhang M, Gu H, et al. An exploration of challenges associated with machine learning for time series forecasting of COVID-19 community spread using wastewater-based epidemiological data. Sci Total Environ. 2023 Feb 1;858(Pt 1):159748. doi:10.1016/j.scitotenv.2022.159748 PMC9597519 36306840

[59] Johnson KE, Vega Yon G, Brand SP, et al. Bayesian generative modeling for heterogeneous wastewater data applied to COVID-19 forecasting. medRxiv [Preprint]. 2026 Feb 23:2026.02.23.26346887. doi:10.64898/2026.02.23.26346887

[60] Tisza M, Javornik Cregeen S, Avadhanula V, et al. Wastewater sequencing reveals community and variant dynamics of the collective human virome. Nat Commun. 2023 Oct 28;14(1):6878. doi:10.1038/s41467-023-42064-1 PMC10613200 37898601

[61] Torabi F, Li G, Mole C, et al. Wastewater-based surveillance models for COVID-19: A focused review on spatio-temporal models. Heliyon. 2023 Nov 1;9(11):e21734. doi:10.1016/j.heliyon.2023.e21734 PMC10694161 38053867

[62] Schoen ME, Bidwell AL, Wolfe MK, et al. United States influenza 2022–2023 season characteristics as inferred from wastewater solids, influenza hospitalization, and syndromic data. Environ Sci Technol. 2023 Dec 12;57(49):20542–20550. doi:10.1021/acs.est.3c07526 PMC10720384 38014848

[63] Wolfe MK, Duong D, Shelden B, et al. Detection of hemagglutinin H5 influenza A virus sequence in municipal wastewater solids at wastewater treatment plants with increases in influenza A in spring, 2024. Environ Sci Technol Lett. 2024 Jun 11;11(6):526–532. doi:10.1021/acs.estlett.4c00331

[64] Lowry SA, Wolfe MK, Boehm AB. Respiratory virus concentrations in human excretions that contribute to wastewater: a systematic review and meta-analysis. J Water Health. 2023 Jun;21(6):831–848. doi:10.2166/wh.2023.057 37387346

[65] Munk P, Brinch C, Møller FD, et al. Genomic analysis of sewage from 101 countries reveals global landscape of antimicrobial resistance. Nat Commun. 2022 Dec 1;13(1):7251. doi:10.1038/s41467-022-34312-7 PMC9715550 36456547

[66] Garner E, Maile-Moskowitz A, Angeles LF, et al. Metagenomic profiling of internationally sourced sewage influents and effluents yields insight into selecting targets for antibiotic resistance monitoring. Environ Sci Technol. 2024 Sep 4;58(37):16547–16559. doi:10.1021/acs.est.4c03726 PMC11411718 39229966

[67] Knight ME, Webster G, Perry WB, et al. National-scale antimicrobial resistance surveillance in wastewater: a comparative analysis of HT qPCR and metagenomic approaches. Water Res. 2024 Sep 15;262:121989. doi:10.1016/j.watres.2024.121989 39018584

[68] Arango-Argoty G, Garner E, Pruden A, et al. DeepARG: a deep learning approach for predicting antibiotic resistance genes from metagenomic data. Microbiome. 2018 Feb 1;6(1):23. doi:10.1186/s40168-018-0401-z PMC5796597 29391044

[69] Tierney BT, Foox J, Ryon KA, et al. Towards geospatially-resolved public-health surveillance via wastewater sequencing. Nat Commun. 2024 Sep 27;15(1):8386. doi:10.1038/s41467-024-52427-x PMC11436780 39333485

[70] Driver EM, Gushgari AJ, Steele JC, et al. Assessing population-level stress through glucocorticoid hormone monitoring in wastewater. Sci Total Environ. 2022 Sep 10;838:155961. doi:10.1016/j.scitotenv.2022.155961 35588803

[71] Baz-Lomba JA, van Nuijs AN, Lenart-Boroń A, et al. Bridging the gap between research and decision making: a European survey to enhance cooperation in wastewater-based epidemiology (WBE) for illicit drugs. Public Health. 2025 Apr;241:158–163. doi:10.1016/j.puhe.2025.02.009 40014941

[72] Dos Santos MC, Silva AC, dos Reis Teixeira C, et al. Wastewater surveillance for viral pathogens: a tool for public health. Heliyon. 2024 Jul 15;10(13):e33873. doi:10.1016/j.heliyon.2024.e33873 PMC11279281 39071684

[73] Child HT, Airey G, Maloney DM, et al. Comparison of metagenomic and targeted methods for sequencing human pathogenic viruses from wastewater. mBio. 2023 Dec 19;14(6):e0146823. doi:10.1128/mbio.01468-23 PMC10746264 37877702

[74] Kantor RS, Jiang M. Considerations and opportunities for probe capture enrichment sequencing of emerging viruses from wastewater. Environ Sci Technol. 2024 May 14;58(19):8161–8168. doi:10.1021/acs.est.4c02638 PMC11097388 38691513

[75] Pagsuyoin S, Ng C, Molejon N, et al. Coupling wastewater-based epidemiology with data-driven machine learning for managing public health risks. Risk Anal. 2025 Jul 6;45(10):2974–2982. doi:10.1111/risa.70075 PMC12516659 40618783

[76] Breiman L. Statistical modeling: the two cultures (with comments and a rejoinder by the author). Stat Sci. 2001 Aug;16(3):199–231. doi:10.1214/ss/1009213726 Available from: http://www.jstor.org/stable/2676681

[77] Karniadakis GE, Kevrekidis IG, Lu L, et al. Physics-informed machine learning. Nat Rev Phys. 2021 May;3(6):422–440. doi:10.1038/s42254-021-00314-5

[78] Pang X, Lee BE, Gao T, et al. Early warning COVID-19 outbreak in long-term care facilities using wastewater surveillance: correlation, prediction, and interaction with clinical and serological statuses. Lancet Microbe. 2024 Oct;5(10):100894. doi:10.1016/S2666-5247(24)00126-5 39182502

[79] Courbariaux M, Cluzel N, Wang S, et al. A flexible smoother adapted to censored data with outliers and its application to SARS-CoV-2 monitoring in wastewater. Front Appl Math Stat. 2022;8:836349. doi:10.3389/fams.2022.836349

[80] Therrien JD, Thomson M, Sion ES, et al. A comprehensive, open-source data model for wastewater-based epidemiology. Water Sci Technol. 2024 Jan;89(1):1–19. doi:10.2166/wst.2023.409 38214983

[81] Cluzel N, Courbariaux M, Wang S, et al. A nationwide indicator to smooth and normalize heterogeneous SARS-CoV-2 RNA data in wastewater. Environ Int. 2022 Jan;158:106998. doi:10.1016/j.envint.2021.106998 PMC8608586 34991258

[82] Aplakidou E, Vergoulidis N, Chasapi M, et al. Visualizing metagenomic and metatranscriptomic data: a comprehensive review. Comput Struct Biotechnol J. 2024 May 3;23:2011. doi:10.1016/j.csbj.2024.04.060 PMC11101950 38765606

[83] Lumley D, Jursic Wanninger D, Magnusson Å, et al. Implementing a digital twin for optimized real-time control of Gothenburg’s regional sewage system. Water Practice & Technology. 2024 Mar 1;19(3):657–670. doi:10.2166/wpt.2024.060

[84] Wang AJ, Li H, He Z, et al. Digital twins for wastewater treatment: a technical review. Engineering. 2024 May 1;36:21–35. doi:10.1016/j.eng.2024.04.012

[85] Allen B. The promise of explainable AI in digital health for precision medicine: a systematic review. J Pers Med. 2024 Mar 1;14(3):277. doi:10.3390/jpm14030277 PMC10971237 38541019

[86] Baethge C, Goldbeck-Wood S, Mertens S. SANRA-a scale for the quality assessment of narrative review articles. Res Integr Peer Rev. 2019 Mar 26;4:5. doi:10.1186/s41073-019-0064-8 PMC6434870 30962953

[87] Roldan-Hernandez L, Van Oost C, Boehm AB. Solid–liquid partitioning of dengue, West Nile, Zika, hepatitis A, influenza A, and SARS-CoV-2 viruses in wastewater from across the USA. Environ Sci: Water Res Technol. 2025;11(1):88–99. doi:10.1039/d4ew00225c

[88] Makridakis S, Spiliotis E, Assimakopoulos V. Statistical and machine learning forecasting methods: concerns and ways forward. PLoS One. 2018 Mar 27;13(3):e0194889. doi:10.1371/journal.pone.0194889 PMC5870978 29584784

[89] Wilkinson MD, Dumontier M, Aalbersberg IJ, et al. The FAIR guiding principles for scientific data management and stewardship. Sci Data. 2016 Mar 15;3:160018. doi:10.1038/sdata.2016.18 PMC4792175 26978244

[90] Ong E, Xiang Z, Zhao B, et al. Ontobee: a linked ontology data server to support ontology term dereferencing, linkage, query and integration. Nucleic Acids Res. 2017 Jan 4;45(D1):D347–D352. doi:10.1093/nar/gkw918 PMC5210626 27733503

[91] He Y, Xiang Z, Zheng J, et al. The eXtensible ontology development (XOD) principles and tool implementation to support ontology interoperability. J Biomed Semantics. 2018 Jan 12;9(1):3. doi:10.1186/s13326-017-0169-2 PMC5765662 29329592

[92] Ceccaroni L, Cortés U, Sànchez-Marrè M. OntoWEDSS: augmenting environmental decision-support systems with ontologies. Environ Model Softw. 2004;19(9):785–797. doi:10.1016/j.envsoft.2003.03.006

[93] Geirhos R, Jacobsen JH, Michaelis C, et al. Shortcut learning in deep neural networks. Nat Mach Intell. 2020 Nov;2(11):665–673. doi:10.1038/s42256-020-00257-z

[94] Hernán MA, Hsu J, Healy B. A second chance to get causal inference right: a classification of data science tasks. CHANCE. 2019;32(1):42–49. doi:10.1080/09332480.2019.1579578

[95] Textor J, van der Zander B, Gilthorpe MS, et al. Robust causal inference using directed acyclic graphs: the R package ‘dagitty’. Int J Epidemiol. 2016 Dec 1;45(6):1887–1894. doi:10.1093/ije/dyw341 28089956

[96] Helsel DR. Fabricating data: how substituting values for nondetects can ruin results, and what can be done about it. Chemosphere. 2006 Dec;65(11):2434–2439. doi:10.1016/j.chemosphere.2006.04.051 16737727

[97] Länsivaara A, Lehto KM, Hyder R, et al. Comparison of different reverse transcriptase–polymerase chain reaction–based methods for wastewater surveillance of SARS-CoV-2: exploratory study. JMIR Public Health Surveillance. 2024 Aug 19;10:e53175. doi:10.2196/53175 PMC11369532 39158943

[98] Augusto MR, Claro IC, Siqueira AK, et al. Sampling strategies for wastewater surveillance: evaluating the variability of SARS-COV-2 RNA concentration in composite and grab samples. J Environ Chem Eng. 2022 Jun 1;10(3):107478. doi:10.1016/j.jece.2022.107478 PMC8882035 35251931

[99] Li J, Ahmed W, Metcalfe S, et al. Impact of sewer biofilms on fate of SARS-CoV-2 RNA and wastewater surveillance. Nat Water. 2023 Mar;1(3):272–280. doi:10.1038/s44221-023-00033-4

[100] van den Berg R, Huijskens E, Groenendijk F, et al. Improving surveillance of infectious diseases in child daycare settings: insights from a pilot study in the Netherlands. Front Public Health. 2026 Apr 10;14:1797334. doi:10.3389/fpubh.2026.1797334 PMC13106512 42040144

[101] Casado-Martín L, Hernández M, González-Peña MJ, et al. A one-year wastewater-based surveillance study of the main human respiratory viruses in a middle-size Spanish city during the COVID-19 pandemic period. Microorganisms. 2026 Jan 9;14(1):151. doi:10.3390/microorganisms14010151 PMC12843686 41597669

[102] Pan SJ, Yang Q. A survey on transfer learning. IEEE Trans Knowl Data Eng. Oct. 2010;22(10):1345–1359. doi:10.1109/TKDE.2009.191

[103] Nguyen DC, Pham QV, Pathirana PN, et al. Federated learning for smart healthcare: a survey. ACM Comput Surv (Csur). 2022 Feb 3;55(3):1–37. doi:10.1145/3501296

[104] Bergmeir C, Benítez JM. On the use of cross-validation for time series predictor evaluation. Inf Sci (Ny). 2012 May 15;191:192–213. doi:10.1016/j.ins.2011.12.028

[105] Roberts DR, Bahn V, Ciuti S, et al. Cross-validation strategies for data with temporal, spatial, hierarchical, or phylogenetic structure. Ecography. 2017 Aug;40(8):913–929. doi:10.1111/ecog.02881

[106] Ganasegeran K, Abdul Rashid S, Kamarudin MK. A narrative review of artificial intelligence applications in wastewater epidemiology. Discover Public Health. 2026;23:699. doi:10.1186/s12982-026-02029-3

[107] Naughton CC, Holm RH, Lin NJ, et al. Online dashboards for SARS-CoV-2 wastewater data need standard best practices: An environmental health communication agenda. J Water Health. 2023 May;21(5):615–624. doi:10.2166/wh.2023.312 PMC10846915 37254909

[108] NASEM (National Academies of Sciences, Engineering, and Medicine). Increasing the utility of wastewater-based disease surveillance for public health action: a Phase 2 Report; 2024 Sep 12. doi:10.17226/27516 39808010

[109] ISO (International Organization for Standardization). Water quality – sampling, detection and quantification of SARS-CoV-2 in wastewater using real-time RT-PCR; 2025. Available from: https://www.iso.org/standard/90137.html

[110] Gitter A, Ruvalcaba V, Clark K, et al. Turning analysis into action: opportunities and challenges in implementing wastewater science for public health decision-making. Front Public Health. 2025 May 21;13:1562659. doi:10.3389/fpubh.2025.1562659 PMC12133736 40469614

[111] Shrestha S, Yoshinaga E, Chapagain SK, et al. Wastewater-based epidemiology for cost-effective mass surveillance of COVID-19 in low-and middle-income countries: challenges and opportunities. Water (Basel). 2021 Oct 15;13(20):2897. doi:10.3390/w13202897

[112] Hamilton KA, Wade MJ, Barnes KG, et al. Wastewater-based epidemiology as a public health resource in low-and middle-income settings. Environ Pollut. 2024 Jun 15;351:124045. doi:10.1016/j.envpol.2024.124045 38677460

[113] Hu X, Keshaviah A, Harrison EB. The costs of wastewater monitoring in low- and middle-income countries. Mathematica. 2023. Available from: https://www.mathematica.org/publications/the-costs-of-wastewater-monitoring-in-low-and-middle-income-countries

[114] Capone D, Chigwechokha P, de Los Reyes FL 3rd, et al. Impact of sampling depth on pathogen detection in pit latrines. PLoS Negl Trop Dis. 2021 Mar 2;15(3):e0009176. doi:10.1371/journal.pntd.0009176 PMC7954291 33651818

[115] Capone D, Chiluvane M, Cumbane V, et al. Environmental pathogen surveillance in cities without universal conventional wastewater infrastructure. medRxiv [Preprint]. 2025 Aug 5:2025.08.01.25332775. doi:10.1101/2025.08.01.25332775 Update in: PLOS Glob Public Health. 2026 Apr 10;6(4):e0004994. doi:10.1371/journal.pgph.0004994 PMC13068267 41961797

[116] Hrudey SE, Silva DS, Shelley J, et al. Ethics guidance for environmental scientists engaged in surveillance of wastewater for SARS-CoV-2. Environ Sci Technol. 2021 Jun 8;55(13):8484–8491. doi:10.1021/acs.est.1c00308 34101444

[117] Holm RH, Anderson LB, Ness HD, et al. Towards the outbreak tail, what is the public opinion about wastewater surveillance in the United States? J Water Health. 2024 Aug 1;22(8):1409–1418. doi:10.2166/wh.2024.074 39212278

[118] Bowes DA, Darling A, Driver EM, et al. Structured ethical review for wastewater-based testing in support of public health. Environ Sci Technol. 2023 Aug 23;57(35):12969–12980. doi:10.1021/acs.est.3c04529 PMC10484207 37611169

[119] Gable L, Ram N, Ram JL. Legal and ethical implications of wastewater monitoring of SARS-CoV-2 for COVID-19 surveillance. J Law Biosci. 2020 Jan;7(1):lsaa039. doi:10.1093/jlb/lsaa039 PMC7337755 32793373

[120] NASEM (National Academies of Sciences, Engineering, and Medicine). Wastewater-based disease surveillance for public health action. Washington (DC): The National Academies Press; 2023. doi:10.17226/26767 37184191

[121] O’Reilly KM, Wade MJ, Farkas K, et al. Analysis insights to support the use of wastewater and environmental surveillance data for infectious diseases and pandemic preparedness. Epidemics. 2025 Jun 1;51:100825. doi:10.1016/j.epidem.2025.100825 40174494

[122] Hidad S, Greeff SC, Haan F, et al. An observational study protocol on wastewater measurements and diaper culture to estimate antimicrobial resistance in long-term care facilities for people with intellectual disabilities: The GIRAF-MIC study protocol. PLoS One. 2025 May 15;20(5):e0324083. doi:10.1371/journal.pone.0324083 PMC12080783 40373105

[123] Gneiting T, Balabdaoui F, Raftery AE. Probabilistic forecasts, calibration and sharpness. J R Stat Soc Ser B Stat Methodol. 2007 Apr;69(2):243–268. doi:10.1111/j.1467-9868.2007.00587.x

[124] Meinshausen N, Ridgeway G. Quantile regression forests. J Mach Learn Res. 2006 Jun 1;7(6):983–999. http://jmlr.org/papers/v7/meinshausen06a.html

[125] Singer AC, Thompson JR, Filho CR, et al. A world of wastewater-based epidemiology. Nature Water. 2023 May;1(5):408–415. doi:10.1038/s44221-023-00083-8

