# Supplementary material for: Machine learning-enabled wastewater-based surveillance for emerging pathogen detection and monitoring: current applications, challenges, and future prospects
Source: Emerg Microbes Infect. 2026 Sep 7;15(1):2731499. doi: 10.1080/22221751.2026.2731499 (PMC13652061; doi:10.1080/22221751.2026.2731499)
Supplement: Supplementary file 1 [file TEMI_A_2731499_SM4857.docx]

**Supplementary materials**

**Supplementary Methods. Literature identification, selection, and classification**

**S.1　Design**

This review is a narrative synthesis. A systematic review protocol was not registered and no formal risk-of-bias instrument was applied, because the aim is to characterize how analytical methods are used across a heterogeneous literature rather than to pool effect estimates. To make the resulting evidence base auditable, the search strategy, the selection criteria, the composition of the cited literature, and the classification applied to analytical sources are reported in full below and in Supplementary Tables S1 to S3.

**S.2　Information sources and search**

PubMed, Web of Science Core Collection, and Google Scholar were searched iteratively between February 24, 2025 and June 30, 2026, with the final update conducted on June 30, 2026. Because the subject spans environmental microbiology, epidemiology, and computational science, the search was not executed as a single fixed string but as four term blocks that were combined and recombined: (i) surveillance setting; (ii) analytical approach; (iii) surveillance target; and (iv) implementation and governance. Blocks (i) and (ii) formed the core query; blocks (iii) and (iv) were used to structure additional searches so that targets other than SARS-CoV-2, and non-technical implementation issues, would not be masked by the volume of COVID-19 literature. The complete term list and a representative executable query are given in Supplementary Table S1. These searches were supplemented by backward and forward citation tracking from key articles and previous reviews, and by targeted consultation of the websites of national and international wastewater surveillance programs and standards bodies (US CDC; European Commission and its Health Emergency Preparedness and Response Authority; WHO; ISO; and the US National Academies of Sciences, Engineering, and Medicine).

**S.3　Eligibility**

Sources were retained when they contributed directly to one of the six roles defined in S.5 and when they reported sufficient methodological detail to be interpreted. Where several publications reported the same dataset or analysis, the most complete version was retained. Screening was restricted to English-language sources. Studies confined to wastewater-treatment process control without a population-health endpoint were not considered, with the exception of two digital-twin studies [83,84] cited specifically to describe the hydraulic and operational simulation layer discussed in Section 4.3.

**S.4　Sources from outside wastewater surveillance**

A second category of sources was drawn deliberately from outside this field. Twenty-one methodological references establish concepts applied throughout the review, including the distinction between statistical and algorithmic modeling cultures, cross-validation under temporal and spatial dependence, substitution bias in censored environmental data, transfer learning and domain adaptation, shortcut learning, causal adjustment sets, and the calibration and sharpness of probabilistic forecasts. These were identified by expert selection rather than by database search, because they are not indexed under wastewater terms. They were included where a methodological point applies to wastewater data by virtue of the data's structure rather than its origin. No claim about demonstrated performance in wastewater surveillance rests on them; each is listed in Supplementary Table S3, Panel B, and where such a source underpins an entry in Table 3 that entry is marked in the table notes.

**S.5　Role assignment**

Each cited source was assigned to one of six roles, which structure the presentation throughout this review: (i) background and scope of WBS; (ii) pathogen detection, monitoring, and other surveillance targets; (iii) analytical evidence, comprising statistical or machine-learning modeling, signal processing, anomaly detection, and dimensionality reduction; (iv) methodological references that define analytical concepts outside the WBS setting; (v) implementation, cost, ethics, and governance; and (vi) program infrastructure, guidance, and standards. Each source is assigned the single role for which it is principally cited; fourteen sources classified under role (ii) also report quantitative analysis as a secondary contribution.

**S.6　Classification of analytical sources**

A source was classified as providing analytical evidence if it fitted a statistical or machine-learning model to wastewater measurements, or quantitatively compared wastewater measurements with an epidemiological indicator or with alternative processing methods. Each such source was assigned one or more of the five analytical tasks used in Section 2, one or more of the four methodological layers defined in Section 1.4, and the surveillance target or targets analyzed. Layers were assigned from the methods actually reported rather than from the authors' own description of their approach as statistical or machine learning; this is why classical statistical models predominate even among sources that describe themselves as machine-learning applications.

**S.7　Evidence grading**

Target-task combinations in Table 2 were graded A, B, or C using the definitions given in that table's notes. Grades were assigned from the quantitative results reported in the cited studies rather than from the authors' self-described limitations, and a source contributed to a grade only for the target and task it actually analyzed. Where a study reported limited, inconsistent, or negative results, it was retained and graded on the same basis as positive findings; such sources are identified in Supplementary Table S2.

**S.8　Grey literature, preprints, and other non-standard sources**

Reviews, guidance documents, dashboards, and other grey literature were deliberately included, but with a restricted role. The cited set comprises four agency reports or guidance documents [3,108,113,120], one international standard [109], and four program webpages or dashboards [12-15]. These were used to describe operational infrastructure, reporting practice, standardization, cost, and governance, and were not treated as empirical evidence of model performance. Web pages were accessed and verified in June 2026; where a page had been archived or relocated, both the archived and the current location are given in the reference list. Two preprints [48,59], one conference proceedings paper [55], and one study protocol without reported results [122] were retained because no peer-reviewed or completed equivalent was available at the time of writing; each is identified as such where it is cited, and none is the sole support for any claim.

**S.9　Limitations of this strategy**

Three limitations follow. First, screening and categorization were performed by the authors without dual independent review, and no formal risk-of-bias assessment was applied; selection therefore reflects our judgment of relevance. Second, restricting screening to English-language sources may under-represent surveillance programs and analytical work reported in other languages, particularly in low- and middle-income settings, which compounds the geographic concentration described in Section 3.3. Third, because the published record over-represents successful applications, the balance of positive to negative findings reported in Supplementary Table S2 should not be read as an unbiased estimate of how often such methods succeed; this is discussed at the start of Section 2.

**Supplementary Table S1. Term blocks, sources searched, and representative query used to identify the literature cited in this review.**

| **Concept block** | **Terms used** | **Sources searched** | **Purpose** |
| --- | --- | --- | --- |
| (i) Surveillance setting | "wastewater-based epidemiology"; "wastewater-based surveillance"; "wastewater surveillance"; "sewage surveillance"; "environmental surveillance" | PubMed/MEDLINE; Web of Science Core Collection; Google Scholar | Core block; applied to all searches |
| (ii) Analytical approach | "machine learning"; "deep learning"; "artificial intelligence"; "random forest"; "gradient boosting"; "neural network"; "forecasting"; "nowcasting"; "anomaly detection"; "normalization"; "data harmonization"; "censored data"; "dimensionality reduction"; "explainable artificial intelligence"; "digital twin"; "federated learning" | PubMed/MEDLINE; Web of Science Core Collection; Google Scholar | Core block; combined with (i) using AND |
| (iii) Surveillance target | "SARS-CoV-2"; "influenza"; "respiratory syncytial virus"; "poliovirus"; "mpox"; "norovirus"; "hepatitis"; "antimicrobial resistance"; "illicit drugs"; "biomarker" | PubMed/MEDLINE; Web of Science Core Collection | Supplementary searches, so that non-SARS-CoV-2 targets were not masked |
| (iv) Implementation and governance | "cost"; "low- and middle-income"; "ethics"; "governance"; "privacy"; "dashboard"; "standardization" | PubMed/MEDLINE; Web of Science Core Collection; Google Scholar | Supplementary searches for Sections 3.3 and 4.1 |
| Citation tracking | — | Reference lists of included articles and of previous reviews; forward citation tracking in Web of Science and Google Scholar | Identification of studies missed by keyword searches |
| Grey literature and standards | — | US CDC; European Commission and HERA; WHO; NASEM; ISO; Mathematica | Description of infrastructure, reporting practice, standards, cost, and governance |
| Methodological references | — | Expert selection from statistics, machine learning, ecology, and epidemiology | Concepts not indexed under wastewater terms (Table S3, Panel B) |

**Representative query (PubMed/MEDLINE, blocks (i) × (ii)):** ("wastewater-based epidemiology"[tiab] OR "wastewater surveillance"[tiab] OR "wastewater-based surveillance"[tiab] OR "sewage surveillance"[tiab] OR "environmental surveillance"[tiab]) AND ("machine learning"[tiab] OR "deep learning"[tiab] OR "artificial intelligence"[tiab] OR "random forest*"[tiab] OR "gradient boosting"[tiab] OR "neural network*"[tiab] OR forecast*[tiab] OR nowcast*[tiab] OR "predictive model*"[tiab])

*Note. Asterisks denote truncation. Searches were iterative rather than a single fixed execution; the table records the term blocks and sources used rather than the yield of one exhaustive query, consistent with the narrative-review design stated in Section 1.5.*

**Supplementary Table S2. Composition of the literature cited in this review (n = 125).**

| **Dimension** | **Category** | **n** | **%** |
| --- | --- | --- | --- |
| Publication type | Primary research article | 80 | 64.0 |
|  | Review | 20 | 16.0 |
|  | Perspective, commentary, or editorial | 12 | 9.6 |
|  | Agency report or guidance document | 4 | 3.2 |
|  | Program webpage or dashboard | 4 | 3.2 |
|  | Preprint | 2 | 1.6 |
|  | Conference proceedings | 1 | 0.8 |
|  | International standard | 1 | 0.8 |
|  | Study protocol (no results reported) | 1 | 0.8 |
| Year of publication | 2001–2009 | 5 | 4.0 |
|  | 2010–2015 | 4 | 3.2 |
|  | 2016–2019 | 12 | 9.6 |
|  | 2020–2021 | 14 | 11.2 |
|  | 2022–2023 | 39 | 31.2 |
|  | 2024–2026 | 51 | 40.8 |
| Role in this review | Background and scope of WBS | 8 | 6.4 |
|  | Pathogen detection, monitoring, and other surveillance targets | 33 | 26.4 |
|  | Analytical evidence (modeling, signal processing, anomaly detection, dimensionality reduction) | 35 | 28.0 |
|  | Methodological reference (concept defined outside the WBS setting) | 21 | 16.8 |
|  | Implementation, cost, ethics, and governance | 15 | 12.0 |
|  | Program infrastructure, guidance, and standards | 13 | 10.4 |
| Within analytical evidence (n = 35) | SARS-CoV-2 alone | 25 | 71.4 |
|  | SARS-CoV-2 among multiple targets | 4 | 11.4 |
|  | Target other than SARS-CoV-2 (AMR determinants, community virome, influenza A, population biomarkers) | 6 | 17.1 |
| Analytical layer applied (n = 35; sources may span more than one layer) | Layer 1 — classical statistical models | 27 | — |
|  | Layer 2 — conventional machine learning | 13 | — |
|  | Layer 3 — deep learning | 2 | — |
|  | Layer 4 — hybrid and mechanistically informed | 3 | — |
| Reported outcome of analytical sources (n = 35) | Positive | 20 | 57.1 |
|  | Mixed or conditional | 9 | 25.7 |
|  | Negative or null | 4 | 11.4 |
|  | Descriptive only | 2 | 5.7 |

*Note. Percentages are of the total cited set (n = 125) except in the final three dimensions, where they are of the analytical-evidence subset (n = 35). Layer assignment follows Section 1.4 and reflects the methods reported rather than the authors' own terminology; Layer 1 predominates because correlation, lag estimation, smoothing, and normalization are the most frequently reported analyses. "Reported outcome" refers to whether a source's own quantitative findings supported the analytical approach it evaluated. Assignment of every individual source is given in Supplementary Table S3.*

**Supplementary Table S3. Panel A. Publication type, assigned role, and citing sections for every source cited in this review**

| **No.** | **Source** | **Publication type** | **Role in this review** | **Section(s) cited** |
| --- | --- | --- | --- | --- |
| 1 | Larsen 2020 | Commentary | Background and scope | 1.1 |
| 2 | Polo 2020 | Review | Background and scope | 1.1 |
| 3 | WHO 2023 | Agency guidance | Infrastructure and standards | 1.1, 1.3, 4.1 |
| 4 | Bowes 2024 | Commentary | Background and scope | 1.1 |
| 5 | Hovi 2012 | Review | Detection and monitoring | 1.1, T1, T2 |
| 6 | Hill 2022 | Commentary | Detection and monitoring | 1.1, T1, T2 |
| 7 | Klapsa 2022 | Research | Detection and monitoring | 1.1, T1, T2 |
| 8 | Medema 2020 | Research | Detection and monitoring | 1.1, T2 |
| 9 | Peccia 2020 | Research | Detection and monitoring | 1.1, T2 |
| 10 | Hamouda 2021 | Review | Background and scope | 1.1 |
| 11 | Fonseca i Casas 2024 | Research | Analytical evidence (SARS-CoV-2) | 2.1, 4.3, T2, T3 |
| 12 | European Commission 2023 | Program webpage | Infrastructure and standards | 1.1 |
| 13 | US CDC 2024 (archived) | Program webpage | Infrastructure and standards | 1.1 |
| 14 | US CDC 2025 | Program webpage | Infrastructure and standards | 1.1 |
| 15 | European Commission 2025 | Program webpage | Infrastructure and standards | 1.1 |
| 16 | Gibas 2021 | Research | Detection and monitoring | 1.1 |
| 17 | Li 2023 | Research | Analytical evidence (SARS-CoV-2) | 1.1, 1.4, 2.1, 2.3, 2.5, T2, T3 |
| 18 | Schenk 2023 | Research | Analytical evidence (SARS-CoV-2) | 1.1, 2.1, 3.2, T2, T3 |
| 19 | Daughton 2020 | Review | Background and scope | 1.1 |
| 20 | Sims 2020 | Review | Background and scope | 1.1, 1.2 |
| 21 | Malla 2024 | Research | Detection and monitoring | 1.1, 1.2 |
| 22 | Parkins 2024 | Review | Background and scope | 1.1, 1.2 |
| 23 | Singh 2024 | Review | Detection and monitoring | 1.1, T1, T2 |
| 24 | Louis 2024 | Research | Detection and monitoring | 1.2, T1, T2 |
| 25 | Zulli 2024 (RSV) | Research | Detection and monitoring | 1.2, T1, T2 |
| 26 | Erster 2022 | Research | Detection and monitoring | 1.2, T1, T2 |
| 27 | Darling 2025 | Research | Analytical evidence (multi-target) | 1.2, 2, 2.3, 3.1, 3.2, T1, T2, T3 |
| 28 | Boehm 2024 | Research | Detection and monitoring | 1.2, T1, T2 |
| 29 | Veneri 2025 | Research | Detection and monitoring | 1.2, T1, T2 |
| 30 | Bowes 2023 | Research | Detection and monitoring | 1.2, T1, T2 |
| 31 | Schenk 2025 | Research | Analytical evidence (SARS-CoV-2) | 2.2, T1, T2, T3 |
| 32 | Ibrahim 2019 | Research | Detection and monitoring | T1, T2 |
| 33 | Zulli 2024 (HAV) | Research | Detection and monitoring | T1, T2 |
| 34 | Rau 2024 | Research | Detection and monitoring | T1, T2 |
| 35 | Kim 2024 | Research | Detection and monitoring | T1, T2 |
| 36 | Yang 2014 | Research | Detection and monitoring | T1, T2 |
| 37 | Hamilton 2018 | Review | Detection and monitoring | T1, T2 |
| 38 | Ajonina 2018 | Research | Detection and monitoring | T1, T2 |
| 39 | Ahmed 2022 | Research | Detection and monitoring | 1.3, 1.4, 3.1, T2 |
| 40 | Chai 2024 | Research | Detection and monitoring | 1.3, 1.4, T2 |
| 41 | Chik 2025 | Research | Analytical evidence (SARS-CoV-2) | 2.3, 3.2, T2, T3 |
| 42 | Maal-Bared 2023 | Research | Analytical evidence (SARS-CoV-2) | 2.3, 3.2, T2, T3 |
| 43 | Holm 2022 | Research | Analytical evidence (multi-target) | 2.3, T2, T3 |
| 44 | Safford 2022 | Research | Analytical evidence (SARS-CoV-2) | 2.3, 3.1, T2, T3 |
| 45 | Lewis-Borrell 2023 | Research | Analytical evidence (SARS-CoV-2) | 2.2, 3.1, T2, T3 |
| 46 | Wade 2022 | Research | Analytical evidence (SARS-CoV-2) | 1.4, 2.2, T2, T3 |
| 47 | Ensor 2024 | Research | Analytical evidence (SARS-CoV-2) | 2.2, T2, T3 |
| 48 | Manuel 2022 | Preprint | Analytical evidence (SARS-CoV-2) | 2.2, T2, T3 |
| 49 | Saravia 2024 | Research | Analytical evidence (SARS-CoV-2) | 2.2, 2.3, T2, T3 |
| 50 | Armenta-Castro 2025 | Research | Analytical evidence (SARS-CoV-2) | 2, 2.2, 2.4, 2.5, 3.3, 4.4, T2, T3 |
| 51 | Lai 2023 | Research | Analytical evidence (SARS-CoV-2) | 1.4, 2, 2.1, 2.2, T2, T3 |
| 52 | Ai 2022 | Research | Analytical evidence (SARS-CoV-2) | 2.1, 2.2, 3.2, 4.4, T2, T3 |
| 53 | Dejus 2023 | Research | Analytical evidence (SARS-CoV-2) | 2.1, 3.2, T2, T3 |
| 54 | Tabassum 2026 | Research | Analytical evidence (SARS-CoV-2) | 2.1, T2, T3 |
| 55 | Ribalta 2023 | Conference proceedings | Analytical evidence (SARS-CoV-2) | 2.1, 4.4, T2, T3 |
| 56 | Tang 2023 | Research | Analytical evidence (SARS-CoV-2) | 2.1, 3.2, 4.3, T2, T3 |
| 57 | Schenk 2024 (mobility) | Research | Analytical evidence (SARS-CoV-2) | 2.1, 4.2, T2 |
| 58 | Vaughan 2023 | Research | Analytical evidence (SARS-CoV-2) | 2, 2.1, 3.2, T2, T3 |
| 59 | Johnson 2025 | Preprint | Analytical evidence (SARS-CoV-2) | 2, 2.1, 2.3, 3.2, 4.3, T2, T3 |
| 60 | Tisza 2023 | Research | Analytical evidence (multi-target) | 1.3, 2.4, 4.2, T2, T3 |
| 61 | Torabi 2023 | Review | Analytical evidence (SARS-CoV-2) | 4.2, T2 |
| 62 | Schoen 2023 | Research | Analytical evidence (influenza A) | T2 |
| 63 | Wolfe 2024 | Research | Detection and monitoring | 2, T2 |
| 64 | Lowry 2023 | Review | Detection and monitoring | 2, T2 |
| 65 | Munk 2022 | Research | Analytical evidence (multi-target) | 1.3, 2.4, T2 |
| 66 | Garner 2024 | Research | Analytical evidence (multi-target) | 1.3, 2.4, T2 |
| 67 | Knight 2024 | Research | Analytical evidence (multi-target) | 4.5, T2 |
| 68 | Arango-Argoty 2018 | Research | Methodological reference | 1.3, 4.5, T2, T3 |
| 69 | Tierney 2024 | Research | Analytical evidence (multi-target) | 4.2, T2 |
| 70 | Driver 2022 | Research | Analytical evidence (multi-target) | 4.5, T2 |
| 71 | Baz-Lomba 2025 | Research | Implementation and governance | 4.5, T2 |
| 72 | dos Santos 2024 | Review | Detection and monitoring | 1.3, 1.4 |
| 73 | Child 2023 | Research | Detection and monitoring | 1.3 |
| 74 | Kantor 2024 | Commentary | Detection and monitoring | 1.3 |
| 75 | Pagsuyoin 2025 | Commentary | Methodological reference | 1.4 |
| 76 | Breiman 2001 | Commentary | Methodological reference | 1.4 |
| 77 | Karniadakis 2021 | Review | Methodological reference | 1.4 |
| 78 | Pang 2024 | Research | Analytical evidence (SARS-CoV-2) | 2.1, 2.2, 4.3, T2, T3 |
| 79 | Courbariaux 2022 | Research | Analytical evidence (SARS-CoV-2) | 2.2, T2, T3 |
| 80 | Therrien 2023 | Research | Infrastructure and standards | 2.3, 4.1, T3 |
| 81 | Cluzel 2022 | Research | Analytical evidence (SARS-CoV-2) | 2.3, T2, T3 |
| 82 | Aplakidou 2024 | Review | Methodological reference | 2.4, T3 |
| 83 | Lumley 2024 | Research | Methodological reference | 4.3, T3 |
| 84 | Wang 2024 | Review | Methodological reference | 4.3, T3 |
| 85 | Allen 2024 | Review | Methodological reference | 3.2, 4.4, T3 |
| 86 | Baethge 2019 | Research | Methodological reference | 1.5 |
| 87 | Roldan-Hernandez 2025 | Research | Detection and monitoring | 2 |
| 88 | Makridakis 2018 | Research | Methodological reference | 2.2 |
| 89 | Wilkinson 2016 | Commentary | Infrastructure and standards | 2.3 |
| 90 | Ong 2017 | Research | Infrastructure and standards | 2.3 |
| 91 | He 2018 | Review | Infrastructure and standards | 2.3 |
| 92 | Ceccaroni 2004 | Research | Infrastructure and standards | 2.3 |
| 93 | Geirhos 2020 | Commentary | Methodological reference | 2.5 |
| 94 | Hernán 2019 | Commentary | Methodological reference | 2.5 |
| 95 | Textor 2016 | Research | Methodological reference | 2.5 |
| 96 | Helsel 2006 | Research | Methodological reference | 3.1 |
| 97 | Länsivaara 2024 | Research | Detection and monitoring | 3.1 |
| 98 | Augusto 2022 | Research | Detection and monitoring | 3.1 |
| 99 | Li 2023 (biofilm) | Research | Detection and monitoring | 3.1, 3.2 |
| 100 | van den Berg 2026 | Research | Implementation and governance | 3.1, 3.2 |
| 101 | Casado-Martín 2026 | Research | Detection and monitoring | 3.1, 3.2 |
| 102 | Pan 2010 | Review | Methodological reference | 3.2 |
| 103 | Nguyen 2022 | Review | Methodological reference | 3.2, 4.2 |
| 104 | Bergmeir 2012 | Research | Methodological reference | 3.2 |
| 105 | Roberts 2017 | Research | Methodological reference | 3.2 |
| 106 | Ganasegeran 2026 | Review | Implementation and governance | 3.2 |
| 107 | Naughton 2023 | Research | Implementation and governance | 3.3, 4.1 |
| 108 | NASEM 2024 (Phase 2) | Agency report | Infrastructure and standards | 3.3, 4.1 |
| 109 | ISO 2025 | International standard | Infrastructure and standards | 3.3, 4.1 |
| 110 | Gitter 2025 | Research | Implementation and governance | 3.3 |
| 111 | Shrestha 2021 | Review | Implementation and governance | 3.3 |
| 112 | Hamilton 2024 | Commentary | Implementation and governance | 3.3 |
| 113 | Hu 2023 | Agency report | Implementation and governance | 3.3 |
| 114 | Capone 2021 | Research | Implementation and governance | 3.3 |
| 115 | Capone 2026 | Research | Implementation and governance | 3.3, 4.2 |
| 116 | Hrudey 2021 | Research | Implementation and governance | 3.3 |
| 117 | Holm 2024 | Research | Implementation and governance | 3.3, 4.5 |
| 118 | Bowes 2023 (ethics) | Research | Implementation and governance | 3.3 |
| 119 | Gable 2020 | Research | Implementation and governance | 3.3 |
| 120 | NASEM 2023 (Phase 1) | Agency report | Infrastructure and standards | 4.1, 4.2 |
| 121 | O'Reilly 2025 | Commentary | Methodological reference | 4.2 |
| 122 | Hidad 2025 | Study protocol | Implementation and governance | 4.2 |
| 123 | Gneiting 2007 | Research | Methodological reference | 4.3 |
| 124 | Meinshausen 2006 | Research | Methodological reference | 4.4 |
| 125 | Singer 2023 | Commentary | Background and scope | 4.5 |

*Note. Sources cited in more than one section are listed under all of them. "T1", "T2", and "T3" denote citations appearing inside Table 1, Table 2, and Table 3. Reference numbers correspond to the final Vancouver-numbered reference sequence in the revised manuscript.*

**Supplementary Table S3. Panel B. Methodological references drawn from outside wastewater surveillance (n = 21)**

| **No.** | **Field of origin** | **Point for which it is cited** | **Where used** |
| --- | --- | --- | --- |
| 68 | Bioinformatics | Deep-learning annotation of antimicrobial resistance genes | 1.3, 4.5, T2, T3 |
| 75 | Risk analysis | Rationale for coupling WBE with data-driven methods | 1.4 |
| 76 | Statistics | Statistical and algorithmic modeling as two cultures | 1.4 |
| 77 | Applied mathematics | Physics-informed learning; digital twins | 1.4 |
| 82 | Bioinformatics | Visualization of meta-omics data for non-specialists | 2.4, T3 |
| 83 | Wastewater engineering | Real-time control twin; dependence on rainfall forecasts | 4.3, T3 |
| 84 | Wastewater engineering | Digital twins for treatment and sewer systems | 4.3, T3 |
| 85 | Digital health | Maturity of explanation methods in health applications | 3.2, 4.4, T3 |
| 86 | Narrative-review methodology | Reporting of literature search under SANRA item 3 | 1.5 |
| 88 | Forecasting | Statistical methods outperforming ML on benchmark series | 2.2 |
| 93 | Machine learning | Shortcut learning and failure of transfer | 2.5 |
| 94 | Epidemiology | Prediction versus causal estimation in variable selection | 2.5 |
| 95 | Epidemiology (software) | Enumeration of minimal sufficient adjustment sets | 2.5 |
| 96 | Environmental chemistry | Substitution for non-detects distorts results | 3.1 |
| 102 | Machine learning | Transfer learning and domain adaptation | 3.2 |
| 103 | Clinical informatics | Federated learning without raw-data exchange | 3.2, 4.2 |
| 104 | Forecasting | Cross-validation for dependent time series | 3.2 |
| 105 | Ecology | Blocked cross-validation for dependent data | 3.2 |
| 121 | Infectious-disease modeling | Framework for deciding when wastewater sampling is informative | 4.2 |
| 123 | Statistics | Sharpness subject to calibration; PIT diagnostics | 4.3 |
| 124 | Machine learning | Quantile regression forests; covariate-dependent intervals | 4.4 |

*Note. No claim about demonstrated performance in wastewater surveillance rests on these sources. Where such a source underpins an entry in Table 3, that entry carries a dagger in the table notes.*

**Supplementary methodological guidance**

The following two tables provide practical methodological guidance that complements Sections 3.1 and 3.2 of the main text. Unlike Supplementary Tables S1–S3, which document literature identification, composition, and source classification, Supplementary Tables S4 and S5 synthesize the implications of censoring and dependence structure for model choice and validation. They are intended as interpretive guidance rather than as independent empirical results.

**Supplementary Table S4. Sensitivity of model families to the handling of censored wastewater measurements.**

| **Layer** | **Model family** | **Principal sensitivity** | **Practical consequence** |
| --- | --- | --- | --- |
| L1 | Censored-data and left-censored dynamic models | Requires that detection and quantification limits be reported | Handles censoring explicitly; performance degrades gracefully as the censored fraction rises |
| L1 | Correlation, smoothing, and trend estimation | Sensitive to the substituted magnitude when substitution is used | Substitution can obscure or reverse an apparent trend; censored-data equivalents are available |
| L2 | Tree ensembles (random forest, gradient boosting) | Insensitive to substituted magnitude, but sensitive to the collapse of all censored values onto one point | The substituted constant may be learned as an informative category rather than as absent information |
| L2 / L3 | Linear, regularized, and neural-network models | Directly sensitive to the substituted magnitude | The choice of substitution value can systematically alter the fitted relationship |
| L3 | Sequence models (LSTM, GRU) | Sensitive to runs of consecutive censored observations | Consecutive non-detects are presented as a genuine low plateau, inflating apparent stability in low-prevalence periods |
| L4 | Semi-mechanistic and Bayesian generative models | Censoring can be represented within the observation model | Uncertainty from censoring can be propagated to the predictive distribution rather than discarded |

*Note. Layers are defined in Section 1.4. Entries describe the mechanism by which censoring affects each model family; they are analytical expectations rather than results of a published empirical comparison, which to our knowledge has not been conducted using wastewater data.*

**Supplementary Table S5. Validation and reporting practices for machine-learning models fitted to wastewater surveillance data.**

| **Aspect** | **Recommended practice** | **Practice to avoid** | **Rationale** |
| --- | --- | --- | --- |
| Temporal partitioning | Rolling-origin or blocked cross-validation; every test period strictly later than its training period | Random k-fold partitioning of the series | Consecutive wastewater observations are near-duplicates; random partitioning measures interpolation rather than forecasting |
| Gap between folds | Insert a buffer between training and test periods where needed to reduce dependence across the training–test boundary | Using adjacent training and test blocks when residual dependence across the boundary is substantial | Residual dependence across the boundary can make test observations overly similar to the training data and lead to optimistic performance estimates |
| Spatial partitioning | Leave-one-site-out or spatially blocked folds; report performance per held-out catchment | Random assignment of sites to folds; pooled performance only | Neighboring catchments share climate, mobility, and often laboratory methods |
| Data-dependent preprocessing | Fit any preprocessing step whose parameters are estimated from the data within the training data only | Data-dependent preprocessing fitted to the full series before partitioning | Data-dependent preprocessing fitted on the full series can leak information from the test period into training |
| Baseline comparison | Report against persistence and seasonal-naive baselines at the stated horizon | Correlation with observed cases reported without a baseline | A model that does not beat persistence has not demonstrated predictive value |
| Horizon reporting | State the forecast horizon and whether any future covariate values were used | Reporting accuracy without specifying the horizon | Accuracy is not comparable across studies unless the horizon is fixed |
| Censoring | Report detection and quantification limits and the proportion of non-detects in training and test periods separately | Reporting a single overall non-detect rate, or none | An apparent drop in performance may reflect a change in the censored fraction rather than poor generalization |
| External validation | Evaluate on an independent catchment or a later season not used in development | Reporting only internally validated performance | Internal validation cannot establish transferability |

*Note. Recommendations follow general principles for evaluating models fitted to temporally and spatially dependent data [104,105]; they are not specific to any single wastewater surveillance program.*
